# Structural and molecular comparison of bacterial and eukaryotic trigger factors

**DOI:** 10.1038/s41598-017-10625-2

**Published:** 2017-09-06

**Authors:** Fabian Ries, Yvonne Carius, Marina Rohr, Karin Gries, Sandro Keller, C. Roy D. Lancaster, Felix Willmund

**Affiliations:** 1 Molecular Genetics of Eukaryotes, University of Kaiserslautern, Erwin-Schrödinger-Str. 70, 67663 Kaiserslautern, Germany; 2 Department of Structural Biology, Saarland University, Center of Human and Molecular Biology (ZHMB), Faculty of Medicine Building 60, 66421 Homburg, Germany; 3 Molecular Biophysics, University of Kaiserslautern, Erwin-Schrödinger-Str. 13, 67663 Kaiserslautern, Germany

## Abstract

A considerably small fraction of approximately 60–100 proteins of all chloroplast proteins are encoded by the plastid genome. Many of these proteins are major subunits of complexes with central functions within plastids. In comparison with other subcellular compartments and bacteria, many steps of chloroplast protein biogenesis are not well understood. We report here on the first study of chloroplast-localised trigger factor. In bacteria, this molecular chaperone is known to associate with translating ribosomes to facilitate the folding of newly synthesized proteins. Chloroplast trigger factors of the unicellular green algae *Chlamydomonas reinhardtii* and the vascular land plant *Arabidopsis thaliana* were characterized by biophysical and structural methods and compared to the *Escherichia coli* isoform. We show that chloroplast trigger factor is mainly monomeric and displays only moderate stability against thermal unfolding even under mild heat-stress conditions. The global shape and conformation of these proteins were determined in solution by small-angle X-ray scattering and subsequent *ab initio* modelling. As observed for bacteria, plastidic trigger factors have a dragon-like structure, albeit with slightly altered domain arrangement and flexibility. This structural conservation despite low amino acid sequence homology illustrates a remarkable evolutionary robustness of chaperone conformations across various kingdoms of life.

## Introduction

The transformation of the one-dimensional genetic information into complex protein structures is a challenging task for cells. Polypeptides that emerge vectorially from translating ribosomes cannot fold completely to the native conformation unless the polypeptide chain is fully synthesized and released from the ribosome. During this process, nascent polypeptides are highly susceptible to premature folding, misfolding, and aggregation^1^. In addition, polypeptides synthesized on cytosolic ribosomes and destined for organelles need to be recruited early and kept in an unfolded state to facilitate their translocation across subcellular membranes^2^. Thus, cells have acquired various factors associating with translating ribosomes to assist protein maturation, to prevent premature folding, and to mediate membrane targeting for translocation^3, 4^. Many of these factors belong to the abundant and structurally diverse family of molecular chaperones, which act at all stages of a proteins life span to promote a balanced protein homeostasis^1^. Across all kingdoms of life, specific molecular chaperones associate with translating ribosomes to guide the initial steps of *de novo* folding of nascent polypeptides^5^. Further downstream, other chaperones take over nascent polypeptides to assist folding and final maturation. In bacteria, the ATP-independent trigger factor (TF) is the predominant chaperone that transiently binds to ribosomes. This association is mediated by the ribosomal protein Rpl23, which positions TF next to the ribosomal exit tunnel for early binding of nascent polypeptides^6^. A comprehensive analysis of TF substrates indicated that the chaperone binds most newly translated polypeptides^7^. In fact, *E*. *coli* cells contain a two- to three-fold molar excess of TF relative to ribosomes, thus providing sufficient chaperones to act on all translating ribosomes^6, 8–10^.

High-resolution structural analyses of various bacterial TF molecules showed that TF adopts a unique elongated conformation resembling a “crouching dragon” with three domains^11^. The N-terminal domain is responsible for ribosome association and contains an essential signature motif for this interaction. This domain further supports the C-terminus to shape the backbone structure of TF. The C-terminal domain is the main module of TF and possesses *in vitro* chaperone activity on its own^12^. This domain folds back to interact with the N-terminus and forms the backbone of TF with an open cavity and two protruding arms. Within the amino acid sequence, both termini are separated by the peptidyl–prolyl *cis*–*trans* isomerase (PPIase) or so-called head domain, which is situated opposite of the N-terminus in the three-dimensional structure. In contrast with other molecular chaperones, which contain one specific substrate binding site, TF seems to use multiple sites across the entire cavity for both hydrophobic and hydrophilic substrate interactions^11^. Thus, a bound substrate is accommodated in a protective environment in the interior of TF to prevent misfolding and aggregation. Various structural data further indicated that the protein is rather flexible, which seems essential for binding of the diverse set of substrates and for ribosome association^6^. Bacterial ribosomes are bound by the monomeric form, while non-ribosome-bound TF shows a fast monomer–dimer equilibrium with a half-life of the dimer of ∼1 s and a KD of ∼1–2  μM^13, 14^. For the function of the TF dimer, opposing data have been reported. On the one hand, it was hypothesized that this dimer serves as an inactive storage form. On the other hand, it was found that such dimers contribute to the stabilization of unfolded substrate species^14–16^.

In eukaryotes, chloroplasts are the only subcellular compartments that appear to contain molecular chaperones of the trigger factor family. Chloroplasts confer photoautotrophy to plants and algae. These organelles contain their own semi-autonomous genome, which encodes for approximately 60–100 proteins of all ~3000 chloroplast proteins. Most of the chloroplast-encoded proteins are major subunits of central protein complexes involved in gene expression and photosynthesis^17^. Since chloroplasts are thought to have evolved from a photosynthetically active cyanobacterium more than a billion years ago, many components of the plastidic gene-expression machinery still resemble their cyanobacterial counterparts^18^. For example, chloroplasts contain 70 S ribosomes each consisting of a small 30 S and a large 50 S subunit^19–22^. Consistently, many components of the chloroplast protein folding and quality control machinery are orthologous to the respective system in prokaryotes^23^. Importantly, the chloroplast gene-expression apparatus also acquired novel mechanisms that are not found in cyanobacteria, which most likely serve the orchestrated expression of the nuclear, mitochondrial, and chloroplast genomes.

While bacterial TF has been studied extensively and might be the best understood molecular chaperone in the literature, no experimental evidence about the function and biophysical properties of plastidic trigger factor exists to date. Here, we describe biophysical and structural properties of plastidic trigger factors from Chlamydomonas (*C*. *reinhardtii*) and Arabidopsis (*A*. *thaliana*). Despite a high sequence variance among different chloroplast trigger factor species, small-angle X-ray scattering (SAXS) experiments indicated that the eukaryotic chaperones have an architecture strikingly similar to their bacterial counterpart. Yet, chloroplast trigger factors also show distinct molecular features that might have evolved to meet the functional requirements of the chloroplasts.

## Results and Discussion

### The chloroplast trigger factor family displays low sequence conservation

**Figure 1:**
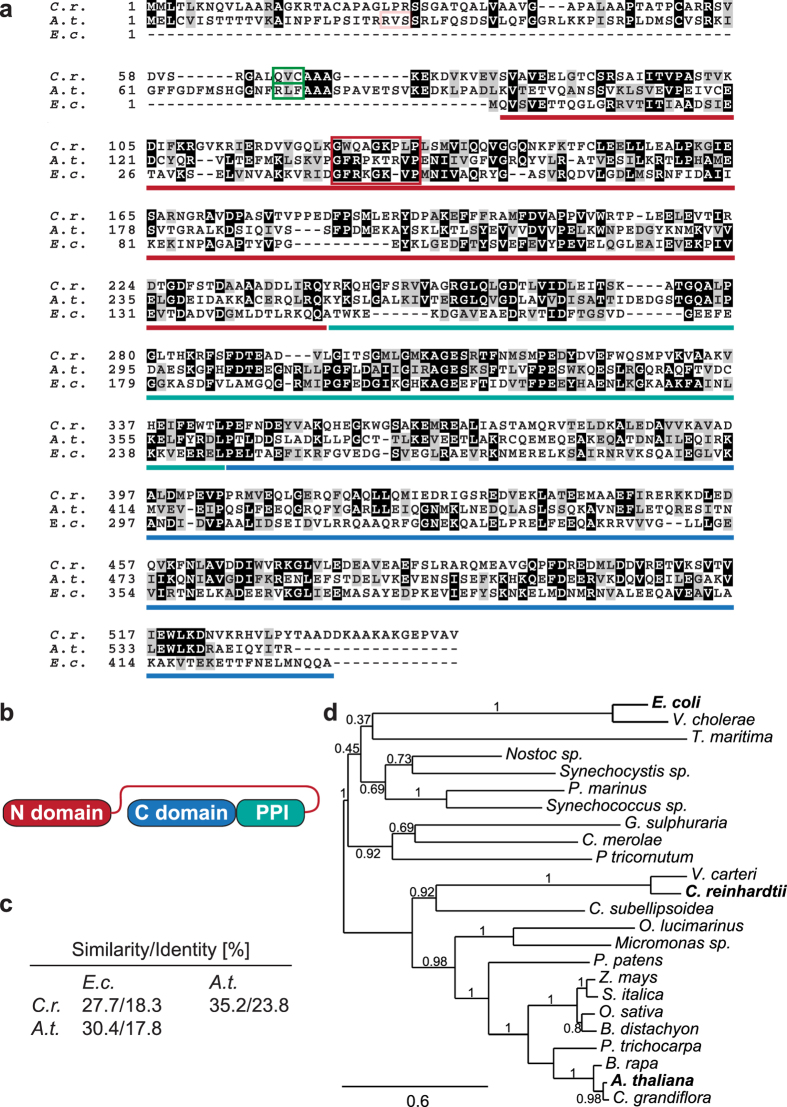
Evolutionary diversity of chloroplast trigger factor. (**a**) Alignment of trigger factor amino acid sequences from *Escherichia coli* (*E*.*c*.), *Arabidopsis thaliana* (*A*.*t*.), and *Chlamydomonas reinhardtii* (*C*.*r*.). Sequences were aligned by ClustalOmega and shaded using BoxShade (http://www.ch.embnet.org). Amino acids highlighted in black are perfectly conserved, similar residues are indicated by a grey background. Boxes indicate chloroplast transit-peptide cleavage sites as determined experimentally (green) and as predicted by TargetP and ChloroP (light red for *At*TIG1)^50, 51^. Dark red box indicates putative ribosome-binding site and lines underneath the sequences indicate the three trigger factor domains according to the *E*. *coli* structure^11^. (**b**) *Cr*TIG1 and *At*TIG1 are predicted to comprise the typical three-domain organization of the N-terminal ribosome-binding domain (red), the PPIase domain (turquois), and the C-terminal domain (blue). (**c**) Sequence homology comparison of *Ec*TF, *Cr*TIG1, and *At*TIG1 according to http://imed.med.ucm.es/Tools/sias.html. (**d**) Phylogram based on amino acid sequence alignments of trigger factor from gram-negative bacteria (*Escherichia coli*, *Vibrio cholerae*, *Thermotoga maritima*), cyanobacteria (*Nostoc sp*. strain PCC 7120, *Synechocystis sp*. strain PCC 6803, *Prochlorococcus marinus*, *Synechococcus sp*. strain WH8102), the diatom (*Phaeodactylum tricornutum*), and the mature TIG1 sequences from red algae (*Galdieria sulphuraria*, *Cyanidioschyzon merolae*), green algae (*Volvox carteri*, *Chlamydomonas reinhardtii*, *Coccomyxa subellipsoidea*, *Micromonas sp*. strain RCC299, *Ostreococcus lucimarinus*), moss (*Physcomitrella patens*), and land plants (*Zea mays*, *Setaria italica*, *Oryza sativa*, *Brachypodium distachyon*, *Populus trichocarpa*, *Brassica rapa*, *Arabidopsis thaliana*, *Capsella grandiflora*). Bootstrap values are given next to the nodes. For sequence information see Supplementary Table S1; for phylogenetic comparison including truncated TIG2 variants see Supplementary Figure S1. The bar indicates branch length.

Genes orthologous to the bacterial TF can be found in all plastid-containing algae and plants but are absent in non-photosynthetic eukaryotes. Thus, it has been postulated that algae and plants encode for a chloroplast isoform^24^. To gain a first understanding of these chloroplast-localised chaperones, we compared plastidic trigger factor proteins from *C*. *reinhardtii* and *A*. *thaliana* with the isoform from *E*. *coli*. For simplicity and consistency with previous studies, the full-length chloroplast proteins will be abbreviated TIG1, and the *E*. *coli* form will be abbreviated *Ec*TF. Excluding the N-terminal chloroplast transit sequence of the TIG1 proteins, chloroplast and bacterial isoforms have a similar overall length and contain the three typical TF domains (i.e., N-terminal ribosome-binding domain, PPI domain, and C-terminal chaperone domain) (Fig. 1a and b). However, the amino acid composition displays only ~18% sequence identity and <30% sequence similarity between the chloroplast TIG proteins and *Ec*TF. Similarly, *Cr*TIG1 and *At*TIG1 also share sequences of only 24% identity and 35% similarity (Fig. 1c). Such low sequence conservation between two phyla is remarkable for molecular chaperones where at least some domains tend to be highly conserved among orthologues^25, 26^. Our phylogenetic analysis of various trigger factor sequences confirmed this high variability among orthologous forms from bacteria, algae, mosses and higher plants. Interestingly, TIG1 orthologues from diatoms and red algae show a closer homology to prokaryotic TFs, while TIG1 proteins from green algae, mosses, and higher plants fall into a separate clade. Within the green lineage, low sequence conservation is observed between chlorophyte and streptophyte TIG1 proteins (Fig. 1d). It might be speculated that the high variety among TIG1s from the green lineage constitutes an evolutionary adaptation of these proteins according to their task during protein biogenesis. Except for the moss *P*. *patens*, all investigated green algal and plant genomes encode only one full-length TIG1 species (Supplementary Table S1). Interestingly, mosses and higher plants seem to encode additional, truncated forms of a trigger factor. We propose to term these orthologous forms TIG2, as transit-peptide analyses predict a chloroplast localisation as well. TIG2 proteins seem to contain only the N-terminal ribosome binding domain. Since the N-terminal domain of bacterial TF is the major site for ribosome binding and exhibits some chaperone activity^6^, an independent or distinct contribution of this truncated variant to protein biogenesis can be envisioned. It has been shown previously by size exclusion chromatography (SEC) and mass spectrometry of *A*. *thaliana* chloroplast extracts that TIG2s are expressed and that they co-migrate in high-molecular weight fractions together with TIG1 and plastid ribosomes^27^. Phylogenetic comparison of TIG1 and TIG2 sequences reveals a clear separation of the two species (Supplementary Figure S1). TIG2 displays a closer relation to the full-length bacterial orthologues compared with TIG1. Thus, it is possible that, during evolution, two trigger factor orthologues were inherited from cyanobacteria and that TIG2 was subsequently lost in algae. Alternatively, TIG2 might have been acquired through horizontal gene transfer after separation of the algal and land-plant lineages.

### Most TIG1 is soluble but also thylakoid-associated

**Figure 2:**
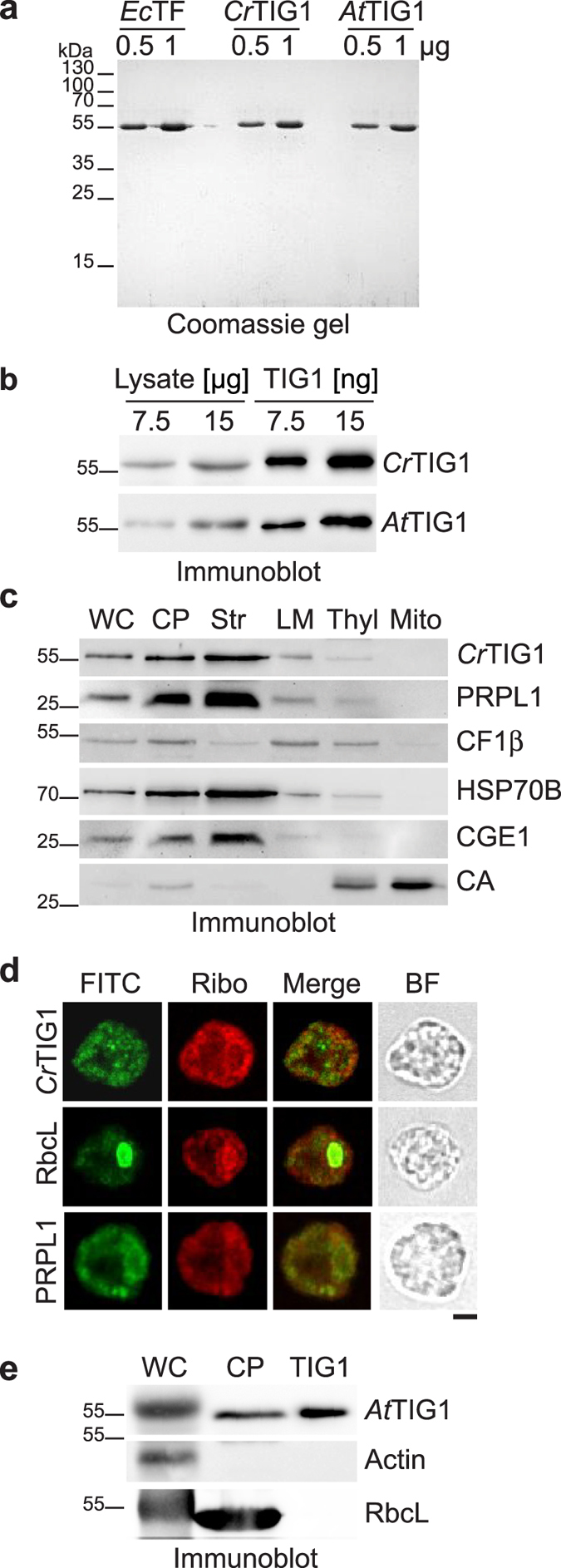
Intracellular localisation of TIG1. (**a**) *Ec*TF, *Cr*TIG1, and *At*TIG1 were heterologously expressed in *E*. *coli*, purified via chitin affinity resins and size exclusion chromatography. 0.5 µg or 1 µg of each protein was separated by SDS-PAGE and stained with Coomassie. (**b**) 7.5 µg or 15 µg of soluble extracts from *C*. *reinhardtii* and *A*. *thaliana* was separated by SDS-PAGE next to 7.5 ng or 15 ng of purified TIG1 protein, transferred to nitrocellulose, and immunoblotted with antibodies against TIG1 (**c**). *C*. *reinhardtii* chloroplasts (CP) were isolated, lysed by hypo-osmotic shock, and separated into stroma (Str), low-density membranes (LM), and thylakoid membranes (Thyl). Mitochondria (Mito) were separated from the same strain. Whole cells (WC) and 7 µg of each fraction were separated by a 7.5–15% SDS-PAGE, transferred to nitrocellulose, and immunoblotted with antibodies against *Cr*TIG1, chloroplast ribosomal protein PRPL1, HSP70B (stroma and membrane control), CF1β (thylakoid membrane associated), stromal CGE1, and mitochondrial carbonic anhydrase (CA). Note that LM might be over-represented compared to thylakoids. (**d**) Images of *C*. *reinhardtii* cells, stained with antibodies directed against *Cr*TIG1, RbcL, and PRPL1 (FITC, green) and a FISH probe against 70 S ribosomes (red). The numbers of cells with the localisation patterns seen was 15 of 20 (75%) for *Cr*TIG1, 16 of 20 (80%) for RbcL, 13 of 20 (65%) for PRPL1, and 20 of 20 (100%) for FISH. Antibody specificity is shown in Supplementary Figure S2. Scale bars represent 2 µm (**e**) 20 µg of soluble *A*. *thaliana* leaf extract or 20 µg of isolated chloroplasts was separated next to 7.5 ng purified *At*TIG1 by SDS-PAGE, transferred to nitrocellulose, and immunoblotted with antibodies against *At*TIG1, Actin, and RbcL. Immunoblots in (**b**, **c**) and (**e**) were cropped to the respective size of the displayed bands.

For a biophysical comparison of chloroplast *Cr*TIG1 and *At*TIG1 with *Ec*TF, mature proteins lacking the N-terminal chloroplast transit peptide were cloned for overexpression and affinity purification followed by subsequent SEC. This yielded highly pure and fully soluble proteins migrating with respective apparent molecular weights of 56 kDa (*Ec*TIG1), 58 kDa (*Cr*TIG1), and 57 kDa (*At*TIG1), which is slightly larger than their theoretical molecular weights (i.e., 48.2 kDa (*Ec*TF), 53.7 kDa (*Cr*TIG1), and 53.2 kDa (*At*TIG1)) (Fig. 2a). To test if the transit-peptide cleavage sites of *Cr*TIG1 and *At*TIG1 proteins were correctly predicted, purified proteins and cell lysates of *C*. *reinhardtii* and *A*. *thaliana* were separated by SDS-PAGE and analysed by immunoblot (Fig. 2b). Both endogenous and purified TIG1 migrated with the same velocity supporting the correct assumptions of transit-peptide cleavage sites.

To confirm that *Cr*TIG1 is indeed a chloroplast-localised protein, we performed subcellular fractionation experiments. *C*. *reinhardtii* cells were fractionated into mitochondria, chloroplasts, stroma, thylakoids, and thylakoid-associated low-density membranes (LMs)^28^. The purity and degree of enrichment were tested with antibodies against chloroplast HSP70B, stromal CGE1, thylakoid membrane-associated CF1β, and mitochondrial carbonic anhydrase (CA). *Cr*TIG1 displayed a distribution similar to the chaperone HSP70B^26^ and was found mainly in the soluble stroma fraction of chloroplasts but is also associated to some extent with LMs and thylakoid membranes (Fig. 2c). Since a major fraction of bacterial TF is ribosome-bound^6^, the distribution of chloroplast ribosomes was visualised with an antibody directed against a protein of the 50 S subunit, PRPL1. Similar to *Cr*TIG1 and HSP70B, ribosomes were enriched mostly in the stromal fraction but were also detectable in the thylakoid fraction. It has previously been reported that in diurnally growing *C*. *reinhardtii* cells, 20–30% of the chloroplast ribosome population is associated with thylakoids^29^. Here, less than 20% of the ribosomes were found in the thylakoid fractions. However, ribosomes may have dissociated from thylakoids during the long preparation process. For an independent assay of *Cr*TIG1 and PRPL1 distribution, *C*. *reinhardtii* cells were examined by immunofluorescence. A hallmark of *C*. *reinhardtii* cells is the single cup-shaped chloroplast, which consumes most of the volume within the cell. The chloroplast contains a globular basal region with the pyrenoid and lobes extending apically from the basal region. Chloroplast localisation of *Cr*TIG1 was confirmed by the typical cup-shape staining (Fig. 2d). Plastidic ribosomes were detected by fluorescence *in situ* hybridization (FISH) against ribosomal RNA and a specific PRPL1 antibody. Comparison of the suborganellar localisations of chloroplast ribosomes and *Cr*TIG1 showed surprisingly different patterns. Ribosomes were mainly found within the globular basal region with highest concentrations proximal to the pyrenoid (highlighted by RbcL staining, Fig. 2d). *Cr*TIG1 staining represented a patchy pattern distributed throughout the chloroplast. A stronger signal was detected in a region which showed little intensity of chloroplast ribosomes and may be outside or at the border of the chloroplast. While it cannot be ruled out that the antibody cross-reacts with cytosolic components, an alternative explanation is that some trigger factor protein may accumulate in a region which was previously shown to contain the cytosolic protein synthesis - and chloroplast import machinery^30^. The enrichment of trigger factor in chloroplasts was independently confirmed in *A*. *thaliana* lysates. Here, immunoblots clearly detect *At*TIG1 in plastid fractions (Fig. 2e).

### Chloroplast TIG proteins are unusually temperature-sensitive

**Figure 3:**
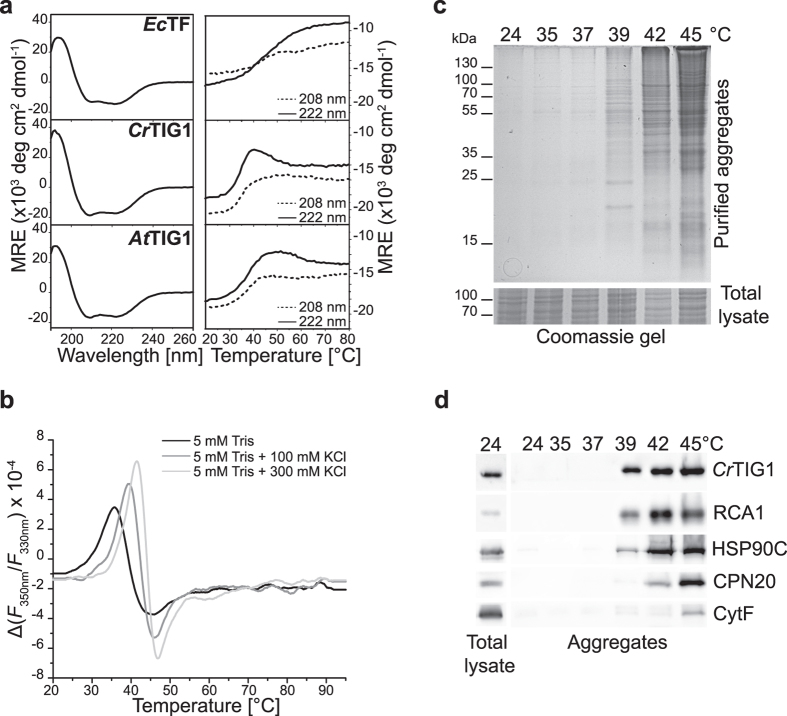
Secondary structure and thermal stability of TIG1s. (**a**) Left panels: CD spectra of 0.1 mg/mL of *Ec*TF, *Cr*TIG1, and *At*TIG1 in 10 mM KCl, 20 mM Tris pH 7.5 buffer were recorded from 190 nm to 280 nm at 20 °C. Right panels: At the two minima at 208 nm and 222 nm, protein-unfolding curves were recorded from 20 °C to 94 °C at a heating rate of 1°/min. (**b**) Thermal stability of purified *Cr*TIG1 was measured at 5 mg/mL with indicated buffers by stepwise heating of 1 °C/min over a temperature range of 20–95 °C. Protein-unfolding midpoints (*T*_m_) were calculated from the first derivative of intrinsic protein fluorescence emission at 330 nm and 350 nm. (**c**) Accumulation of insoluble protein aggregates was observed upon cell exposure to various temperatures of heat stress. Protein aggregates and total lysates were separated by 12% SDS-PAGE and visualized by Coomassie staining. Gel slice of total lysates serves as input control. (**d**) Cropped images of immunoblots of samples shown in (**c)**. show the accumulation of *Cr*TIG1, Rubisco activase (RCA1), HSP90C, CPN20, and Cytochrome F in protein aggregates.

The secondary structure of *Ec*TF is predominantly α-helical for the N- and C-terminal domains, whereas the PPIase mainly contains β-sheets^11, 31^. To gain insight into the secondary structures of the chloroplast TIG1 isoforms, both mature proteins were analysed by far-UV circular dichroism (CD) spectroscopy and compared with *Ec*TF. Consistent with previous reports^31, 32^, the far-UV CD spectrum of *Ec*TF contained two pronounced minima at 208 nm and 222 nm, both typical for α-helical secondary structure. The spectra of both plastidic TIG1 proteins were comparable to that of *Ec*TF, indicating that their overall secondary structures are similar (Fig. 3a). Secondary-structure estimation by the K2D2 algorithm^33^ indicates ~40% α-helix and ~13% β-sheet content for *Ec*TF, close to the 43% α-helical and 16% β-sheet content determined from the respective crystal structure^11^. Both plastidic TIG1 proteins seem to contain a slightly higher proportion of α-helices (~44%), as indicated by a more intense negative ellipticity at 208 nm as compared with *Ec*TF, and a similar content of β-sheets (12% and 13%, respectively). The data also confirm that all heterologously produced and purified proteins were folded and thus suitable for subsequent *in vitro* assays.

Thermal unfolding was studied by gradual heating of the proteins (Fig. 3a, right panels). The transition curves at 208 nm and 222 nm revealed qualitative differences between the bacterial and the plant proteins. For *Ec*TF, a major unfolding transition was only observed at 222 nm, which started at > 30 °C and had a midpoint (where half of the population is folded and the other half unfolded) between 45 ° and 50 °C. This is consistent with another study^34^ using differential scanning calorimetry, which determined the unfolding midpoint of *Ec*TF to be at 54 °C. This resistance against thermal unfolding was attributed to the dimeric form of *Ec*TF^34^. For the chloroplast proteins, unfolding was traceable at both wavelengths, with unfolding starting just above 30 °C and featuring a local maximum in the ellipticity recorded at 222 nm at intermediate temperatures. The midpoints of the thermal unfolding of *Cr*TIG1 and *At*TIG1 were between 35 °C and 40 °C, which are far below the values for *Ec*TF (Fig. 3a, right panels). Thus, even mild heat-stress conditions^35^ seem to cause partial unfolding of at least some domains. Such conformational changes may inactivate the chaperone or reprogram its function for other tasks during heat stress. For a more precise determination of the unfolding midpoint of *Cr*TIG1, shifts of intrinsic tryptophan fluorescence upon thermal unfolding were recorded at wavelengths of 330 nm and 350 nm. We determined the unfolding midpoints *T*_m_ in buffers having various ionic strengths of 0–300 mM KCl. With no additional salt present, *Cr*TIG1 showed a *T*_m_ of 35.6 °C, whereas *T*_m_ was shifted up to 41.6 °C at 300 mM KCl (Fig. 3b). Thus, more physiological salt concentrations render the protein more robust against thermal unfolding.

We next tested if thermal unfolding could also be observed in living cells, which might lead to the accumulation of *Cr*TIG1 in protein aggregates. For this purpose, *C*. *reinhardtii* cells were exposed to various temperatures of mild to severe heat stress (i.e., 35–45 °C), and insoluble aggregates were isolated (see Methods). Overall, little aggregation was observed in cells exposed to temperatures between room temperature and 37 °C. At non-lethal heat stress between 39 °C and 42 °C^35^, increasing protein aggregation was observed with rising temperatures (Fig. 3c). The onset of protein aggregation at 39 °C was confirmed by examining the known heat-labile chloroplast protein rubisco activase (RCA1)^36^, which started to accumulate in aggregates at temperatures ≥39 °C. Interestingly, *Cr*TIG1 was also detectable in the same fractions as RCA1 (Fig. 3d), while other chloroplast chaperones such as HSP90C or the co-chaperonin CPN20 predominantly accumulated in aggregates at higher temperatures. Thus, in contrast to bacterial TF and other molecular chaperones, chloroplast TIG1 is a remarkably heat-sensitive chaperone, which accumulates in aggregates even under relatively mild heat-stress conditions. Unlike many other chaperones, trigger factor is an ATP-independent chaperone. Thus, the observed heat sensitivity may serve as passive regulator to target this chaperone to protein aggregates which may serve as protective mechanism to rescue unfolded proteins from aggregates upon return to physiological growth conditions.

### Chloroplast TIG1 is mainly monomeric

**Figure 4:**
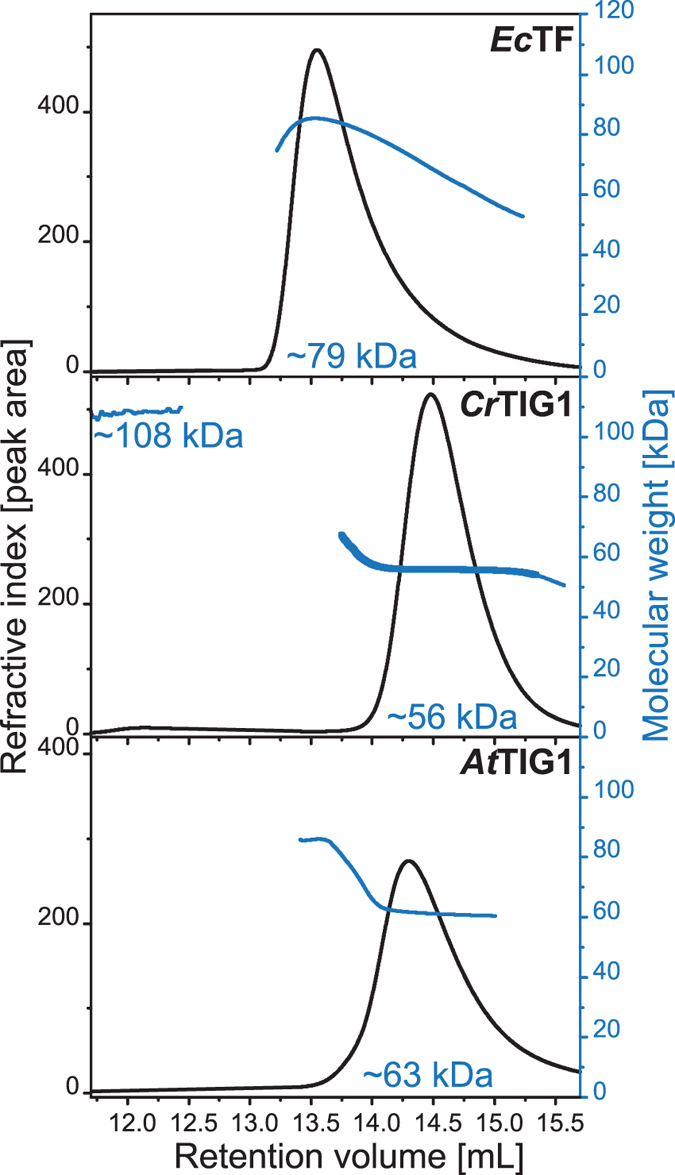
Purified chloroplast TIG1 is mainly monomeric. Determination of conformational states of *Ec*TF, *Cr*TIG1, and *At*TIG1. 200 µg of protein was separated by SEC in a buffer containing 20 mM Tris pH 7.5 and 150 mM KCl and measured with a right-angle light scattering (RALS) detector combined with a refractive index (RI) detector. Here, the RI detector signal was used to follow the elution. Average molar masses are indicated in blue for each graph (right ordinate). For the full elution profile, see Supplementary Figure S3.

Several structural studies reported the occurrence of dimeric *Ec*TF both *in vitro* and *in vivo* ^14, 32, 37^. To determine if chloroplast TIG1s show similar dimerization behaviours, purified TIG1 protein was separated by SEC. A prominent peak of *Ec*TF protein eluted at a volume of 13.6 mL (Fig. 4, top panel), while *Cr*TIG1 eluted in a minor peak at high molecular weight (12.1 mL) and a major peak at 14.5 mL (Fig. 4, middle panel), and *At*TIG1 eluted at 14.1 mL with a small shoulder preceding the actual peak (Fig. 4, bottom panel). Since SEC elution profiles are not precise enough to determine the molecular weights of particles that are clearly nonspherical, as expected at least for *Ec*TF, each fraction of the elution peak was examined online by right-angle static light scattering (RALS). The sloping curve of average molecular weight for *Ec*TF resembles the molecular weight distribution of an earlier study^14^ with lower apparent masses of the protein at the peak borders and the highest value of 79 kDa in the centre of the peak (Fig. 4, top panel, Table S2). As stated in earlier studies, a heterogeneous population of monomeric and oligomeric *Ec*TF seems to co-elute within this peak, which might represent a fast equilibrium of monomeric and oligomeric states^14^. By contrast, both *Cr*TIG1 and *At*TIG1 were monodisperse, as indicated by the consistent molecular weight values within the major peak area (Fig. 4, centre and bottom panel) with polydispersity values of 1.001 and 1.002 (*M*_*w*_ */M*_*n*_), respectively. Further, elution profiles resulted in apparent molecular weight values close to their theoretical monomeric masses (Supplementary Table S2). Hence, at the physiological salt conditions used here, chloroplast TIG1s appeared to be present mainly in a monomeric form. In earlier proteomic studies, *At*TIG1 migrated in native PAGE as expected for a molecular weight of the dimeric state^38^. Dimeric species of TIG1 proteins cannot be excluded under certain conditions and in a native context; however, asymmetric and elongated proteins are known to migrate slower than globular proteins in native gels^39^, which might explain the higher apparent molecular weight of *At*TIG1 in the earlier studies. For our subsequent assays, it was essential that SEC fractions of *Cr*TIG1 and *At*TIG1 be highly monodisperse, making them ideally suited for three-dimensional bead modelling by SAXS.

### SAXS reveals a typical dragon-shape conformation of TIG1

**Figure 5:**
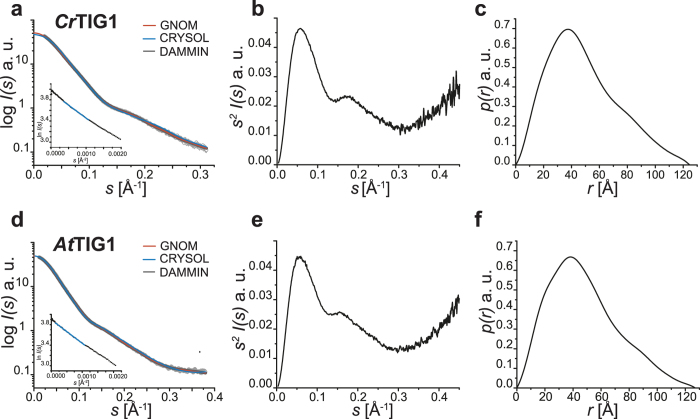
SAXS data from static measurements of trigger factors. Small-angle X-ray scattering data were collected for various concentrations of TIG1 proteins and merged. (**a**) and (**d**) Experimental SAXS profiles from merged scattering curves of *Cr*TIG1 and *At*TIG1 are indicated by small circles. Respectively, red curves represent the fit by *GNOM*, blue curves the best *CRYSOL* fit, grey curves the theoretical scattering of final *DAMMIN* models. Inset, Guinier plot of ln *I*(*s*) *versus s* ^*−*2^ obtained from *AUTORG*. Data points used by *AUTORG* are labelled in blue. (**b**) and (**e**) Kratky plot *s* ^2^ *I*(*s*) *versus s* ^−1^ of *Cr*TIG1 and *At*TIG, respectively. (**c**) and (**f**) Corresponding *p*(*r*) function as calculated from the experimental scattering curves using *GNOM*. “au” is arbitrary units. For SAXS data on *Ec*TIG and the *FoXS* fit, see Supplementary Figure S4.

SAXS was used to examine if chloroplast TIG1s possess a similar overall shape as the bacterial isoform or if the eukaryotic chaperones evolved other structural features. Initial static measurements were performed at different protein concentrations in three replicates to examine if the TIG1 proteins remain monodisperse also at higher protein concentrations. Importantly, SAXS parameters of the bacterial *Ec*TF were very comparable to previously published datasets (Supplementary Figure S4)^32^. At the concentrations used here, the monomer–dimer equilibrium of *Ec*TF was strongly shifted towards the dimeric state, although proteins are known to quickly fluctuate between monomeric and dimeric states even at high concentrations^14^. Indeed, the values of the Porod volume indicate mainly dimeric proteins in the samples, but the molecular weight values derived from *I*(0) (i.e., the intensity at zero angle) point to a fast fluctuating population (Supplementary Table S3). Further, it has been reported that the radius of gyration (*R*_g_) and the maximal particle size (*D*_max_) of *Ec*TF decrease with increasing protein concentration, possibly because of a more densely packed dimer at higher concentrations compared with the monomer at lower concentrations^32^. Consistently, the *R*_g_ and *D*_max_ values determined here also decreased with increasing protein concentration, albeit to a lesser extent (Supplementary Table S3). Chloroplast *Cr*TIG1 and *At*TIG1 appeared predominantly as monomers assuming a predicted molecular weight of 53.7 kDa and 53.2 kDa, respectively. In contrast to *Ec*TF, molar masses calculated from the *I*(0) values slightly increased with rising protein concentrations, most likely because of weak interparticle interference (Supplementary Tables S4 and S5). However, the high linearity of the Guinier plots speaks against aggregation of the samples (Fig. 5a and d, inset)^40^. To overcome this interference, low-range values at low concentrations and high-range values at high concentrations were merged for further processing. Both plastidic proteins were found to have an *R*_g_ value of ~38 Å, as determined by the Guinier plot and *p*(*r*) function. This very large *R*_g_ for a 53-kDa protein—as compared to an *R*_g_ value of 29.9 Å of globular BSA^41^—indicates an elongated and non-globular shape of both proteins. This notion is supported by the asymmetric peak of the pair distance distribution functions of both TIG1s with a shoulder extending to a maximum particle size of 125 Å for *Cr*TIG1 and 127 Å for *At*TIG1, respectively (Fig. 5c and f).

All analysed trigger factor proteins showed an overall similar bimodal pattern in Kratky plots with a dominant first peak followed by a distinct small peak indicating well-folded and multi-domain proteins. In addition, pronounced intrinsic flexibilities are indicated by the behaviour of the plots at higher *s*-range, which do not converge to the abscissa. The Kratky plot of *Ec*TF displays a slight shift compared with both plastidic TIG1 proteins, indicating a more rigid and compact conformation due to the dimeric state^32^ (Fig. 5b,e and Supplementary Figure S4).

**Figure 6:**
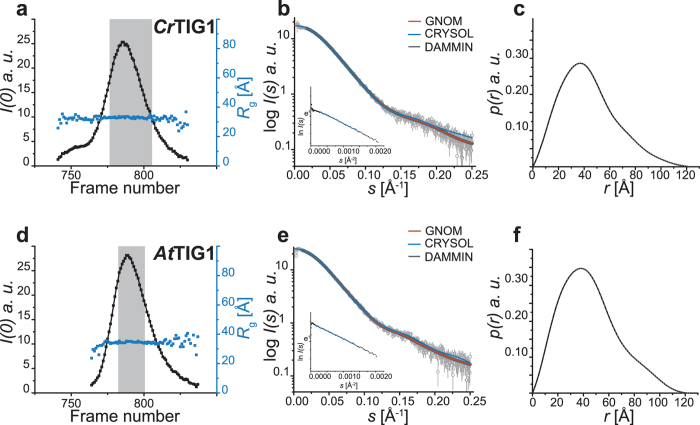
SAXS data from HPLC-SAXS experiments of trigger factors. Small-angle X-ray scattering data were collected by size-exclusion high-performance liquid chromatography with a ENrich SEC 650 column online with small-angle X-ray scattering (HPLC-SAXS) with 400 µg of the respective protein. (**a**) and (**d**) Elution profiles of SEC-SAXS runs, represented by *I*(0) and *R*_g_ determined by *AUTORG* for each frame. Highlighted in grey are the frames used for averaging. (**b**) and (**e**) Experimental SAXS profiles from the averaged HPLC-SAXS frames of *Cr*TIG1 and *At*TIG1 are indicated by small circles. Respectively, red curves represent the fit by *GNOM*, blue curve the *CRYSOL* fit of the RaptorX models, and grey curves the theoretic scattering of final *DAMMIN* model. Inset: Guinier plot of ln *I*(*s*) *versus s* ^*−*2^ obtained from *AUTORG*. Data points used by *AUTORG* are labelled in blue. (**c**) and (**f**) Corresponding *p*(*r*) function as calculated from the experimental scattering curves using *GNOM*. “au” is arbitrary units. For SAXS data on *Ec*TF and the *FoXS* fit, see Supplementary Figure S5.

To verify our results regarding the monomeric state obtained from static SAXS measurements, we further performed online SEC coupled to SAXS detection (HPLC-SAXS). Consistent with the static assays, only minor amounts of putative dimer populations were detectable in the *Cr*TIG1 and *At*TIG1 samples, as indicated by the slight tailing of the monomeric peak fraction (Fig. 6a and d). To simplify our analysis of the chloroplast proteins, these higher molecular weight fractions were neglected in all subsequent steps. Thus, only frames within the peak section of high quality and constant *R*_g_ were averaged for further analysis (Fig. 6a,d and Supplementary Figure S5, grey shaded area). The molecular weight was determined using multiple, concentration-independent, methods. These seem to overestimate the mass of the chloroplast TIG1s by up to 17% (Table 1), which might be attributed to traces of dimers or to the influence of capillary fouling on scattering^42^. However, the masses determined by RALS strongly supported predominantly monomeric states. Importantly, the calculated *R*_g_ (34.4 Å for *Cr*TIG1, 35.5 Å for *At*TIG1) and *D*_max_ (122 Å for *Cr*TIG1, 124 Å for *At*TIG1) values were comparable to the results from static measurement, which allowed us to complementarily process data from both approaches for further modelling.

**Table 1:** Structural parameters derived from static and HPLC-SAXS experiments.

	Protein	*I*(0)	*R*_*g*_ [Å]	Quality [%]	*I*(0)	*D*_max_ [Å]	*R*_*g*_ [Å]	V*p* [Å^3^]	MW [kDa] V*p*	MW [kDa] *I*(0)_BSA_	MW [kDa] *I*(0)_Water_	MW [kDa] datmow	MW [kDa] (*DAMMIN*)
		based on Guinier fit			calculated from *p*(*r*) function								
Static SAXS	*Ec*TF	75.48 ± 0.05	37.9 ± 0.1	89	75.76	132	38.5	169080	99	75	73	107	n.d.
	*Cr*TIG1	49.11 ± 0.06	37.2 ± 0.1	89	49.06	127	38.7	103370	61	49	47	77	62
	*At*TIG1	51.60 ± 0.09	38.3 ± 0.8	78	50.96	125	38.5	115770	68	51	50	72	67
HPLC SAXS	*Ec*TF	—	37.4 ± 0.1	90	—	127	37.9	190000	111	—	—	108	107
	*Cr*TIG1	—	33.3 ± 6	81	—	122	34.4	123140	72	—	—	65	67
	*At*TIG1	—	34.5 ± 0.3	87	—	124	35.5	119000	70	—	—	67	66

For static measurements, scattering curves from different concentrations were merged. For HPLC-SAXS, parameters were determined for the averaged peak scattering. Molecular weights (MW) were estimated using *Porod-volume*/1.7, the *DAMMIN* volume/2, and using the *datmow* tool included in the *ATSAS* suite.

All plastidic TIG1 SAXS datasets were applied for *ab initio* modelling of low-resolution bead envelopes. Modelling of *Ec*TF was not pursued with our dataset since the heterogeneous population of dimeric and monomeric species complicates modelling and different conformations of purified *Ec*TF proteins were comprehensively studied before^32^. With the static SAXS measurements, more defined scattering at a higher *s*-range was obtained because of a higher protein concentration in the sample. Hence, *ab initio* modelling was pursued with the *GASBOR* algorithm, which allows a chained assembly of dummy residues corresponding to the number of amino acids. The models obtained fitted the experimental data with χ^2^ values of 0.81 for *Cr*TIG1 and 0.69 for *At*TIG1 (Supplementary Table S6). To confirm that the minor traces of putative dimeric species did not significantly influence the models, data were processed in symmetry P1 (no symmetry) and P2 (dimer). The P2 symmetry could be excluded because of the resulting high χ^2^ values of these models (data not shown) and hence supported the fact that our models were truly based on the monomeric molecules. For the HPLC-SAXS dataset, *ab initio* modelling was performed with *DAMMIF*. Consistent with the modelling of the static SAXS data, χ^2^ values of 0.72 for *Cr*TIG1 and 0.79 for *At*TIG1 (Supplementary Table S6) were gained for these low-resolution structures.

**Figure 7:**
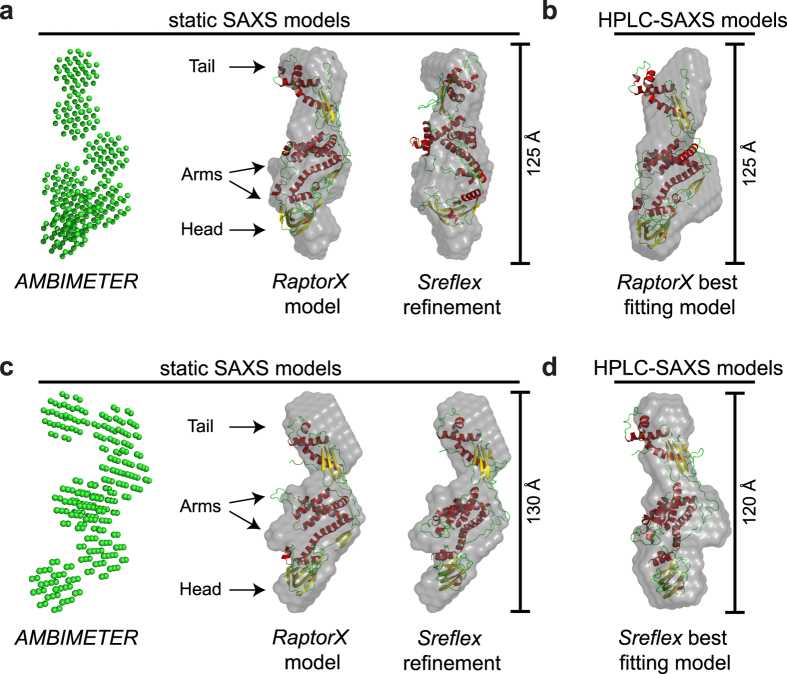
Global shape of the chloroplast trigger factor. *Ab initio* modelling from SAXS data. Shape topology and low-resolution bead models of *Cr*TIG1 (**a**,**b**) and *At*TIG1 (**c**,**d**) from static SAXS experiments (**a**,**c**) and HPLC-SAXS (**b**,**d**) were generated using *AMBIMETER* and *DAMMIN* based on 10 averaged *GASBOR* models for static or 20 *DAMMIF* models for HPLC-SAXS experiments, respectively. Bead models were superimposed by *SUPALM* on the RaptorX high-resolution model or the improved models by *SREFLEX*. Secondary structures were coloured in yellow (β-sheets), red (α-helix), and green (random coils). For different side views of the models, see Supplementary Figure S6.

The low-resolution bead models of *Cr*TIG1 and *At*TIG1 proteins resulting from both datasets clearly resemble the conformation of the bacterial TF with a dragon-like structure containing head, tail and arms (Fig. 7). Interestingly, groove-like structures are visible along the middle domain (Supplementary Figure S6), which could constitute putative binding sites for peptide stretches of chloroplast TIG1 substrates.

Next, we attempted to fit high-resolution models into the SAXS-based *ab initio* shapes. Since, to our knowledge, no structural data exist for any chloroplast trigger factor to date, predicted models were calculated with the RaptorX^43^ and *SAXSTER* ^44^ algorithms on the basis of various structural data of exclusively full-length bacterial isoforms. Different high-resolution structures of TF exist for dimeric apo *E*. *coli* TF complexes (X-ray structure, PDB entry 1w26^11^) and monomeric TF associated with different fragments of the PhoA (NMR structures, PDB entries 2mlx/2mly/2mlz^37^) or the ribosome (PDB entry 2vrh^12^). Further, crystal structures of dimeric TF from *V*. *cholerae* (PDB entry 1t11^45^) and *T*. *maritima* are available. For the latter, both full-length structures with and without a substrate are monomeric (X-ray structures, PDB entries 3gty/gt0^46^). Different bacterial TF species show various domain orientations^47^. For example, both the crystal structure and NMR structural analyses reveal different orientations of the head domain among *Ec*TF^47^, *Vc*TF^44^, and *Tm*TF^46^. For *Vc*TF, both domains constituting the arms and the tail show a twist, which is not seen for the other isoforms^45^. Psi-Blast searches with sequences of *Cr*TIG1 and *At*TIG1 resulted in similar matches between the plastidic TIG1 proteins and the bacterial TFs (Supplementary Table S7). The best coverage for *Cr*TIG1 and *At*TIG1 was reached with the full-length NMR and cryoEM structures of *Ec*TF (PDB entry 2mlx and 2vrh) (Supplementary Table S7). Our predicted models were validated with *CRYSOL* and superimposed with the bead models. Again, the models that gave the best χ^2^ values in *CRYSOL* were the predicted models from RaptorX based on 2mlx. Importantly, not all high-resolution models fitted with same quality. Superimposing the models based on *Vc*TF and *Tm*TF to the SAXS data was not successful as judged by their high χ^2^ scores (>25–55, data not shown). To test if we could further improve the domain agreement of our positive models within the SAXS-based bead models, *SREFLEX* was applied, which examines the flexibility of models. Generally, this refinement improved the χ^2^ values, albeit with a different outcome. For *Cr*TIG1, χ^2^ values were slightly improved from 10.4 to 6.2. *SREFLEX* Refinement of *Cr*TIG1 might have been less than optimal since the orientations of some α-helices within the central body domain were severely altered (Fig. 7a). In contrast, the refinement of *At*TIG1 high-resolution models improved χ^2^ values significantly from 57.2 to 3.3 (Supplementary Table S6) with only mild rearrangements within the domains (Fig. 7c). As shown in Figs 5 and 6, two independent evaluations of our high-resolution models by *CRYSOL* and a simulation of theoretical scattering and fitting to our experimental data by *FoXS* resulted in close fits of the models to the SAXS data. Thus, it can be assumed that the *E*. *coli*-based models closely describe the overall shape and domain position of the plastidic TIG1 proteins. However, none of the bacteria-derived models tested resulted in a complete match to the SAXS shapes obtained from the static and HPLC- SAXS measurements. This suggests that the intrinsic fold of the chloroplast TIG1 proteins, especially the relative arrangement of the three domains to each other, may be unique for TIG1s (Fig. 7). This is underscored by the relatively high normalized spatial discrepancy values (NSD, a measure of quantitative similarity between sets of three-dimensional points), which are larger than 2.5 for all *SREFLEX*-refined models of plastidic TIG1s (Supplementary Table S6). These differences seem most pronounced for the arms, which are further away in both chloroplast TIG1 conformations compared with *Ec*TF^11^, and the head and tail domains, which cannot be described perfectly by our models (Fig. 7 and Supplementary Figure S6). To fully resolve these altered conformations of chloroplast trigger factor, atomic structures are required to determine how chloroplast TIG1 is tailored to perform its function within the chloroplast.

## Conclusions

In conclusion, we present here the first description of two eukaryotic chloroplast-localised molecular chaperones of the trigger factor family. Both plastidic TIG1 orthologues are mainly monomeric, as shown independently by RALS and SAXS for the purified proteins. The most surprising finding was the high propensity of plastidic TIG1 for thermal unfolding, which might perform a specific task during heat exposure. Independent static and HPLC SAXS measurements and subsequent *ab initio* modelling revealed the typical dragon-shape of the molecule as known from bacterial TF containing a head, a tail, and two arms. This trigger-factor-like shape was confirmed by independent high-resolution models, which fitted well to the SAXS data. However, the chloroplast TIG1 models indicate unique domain arrangements and intrinsic conformations, thus suggesting that plastidic TIG1s have structurally diverged from their bacterial orthologues. Given the ubiquitous action of bacterial TF on most newly-synthesized proteins, plastidic TIG1 – and maybe even the truncated version TIG2 – may have specifically adapted to serve the maintenance of protein homeostasis in chloroplasts. It will be interesting to see why chloroplasts are the only compartment of eukaryotic cells that conserved these trigger factor chaperones. Future studies are also required to determine the detailed function and substrate specificity of these chaperones in chloroplasts.

## Methods

### Cells and Culture Conditions

Most experiments were conducted with the *C*. *reinhardtii* strain cw15 CF185^48^. Microscopy was done with strain cw15–325 (cw_d_, mt^+^, arg7^−^). All cultures were grown photomixotrophically in TAP medium^49^ on a rotary shaker at 25 °C and an illumination of 70 E m^−2^ s^−1^. For chloroplast isolation, cells were grown in TAP medium supplemented with 0.5% (*w/v*) peptone. For *A*. *thaliana* cell extracts, plants were grown under standard conditions.

### Cloning, production and purification of the trigger factor species

For *Cr*TIG1 both TargetP and ChloroP^50, 51^ predicted the transit peptide cleavage site QVC/A. Mature *C*. *reinhardtii* TIG1 protein (lacking the putative N-terminal 67 amino-acid transit peptide) was created by site directed mutagenesis of pFW13 resulting in pFW115. For *At*TIG1, the predicted cleavage site was RVS/S. Mature *A*. *thaliana* TIG1 (lacking the putative N-terminal 27 amino-acid transit peptide), was amplified by PCR from cDNA with the primers (5′-GGGGCATATGTCCCGTCTCTTCCAATCAG-3′) and (5′-CCCCGGATCCTCAACGAGTGATGTATTGAATC-3′) and cloned with NdeI and BamHI into pTyb21 (NEB) resulting pFW141. Since the cleavage site was predicted incorrectly, the cleavage site RLF/A was reassigned based on the alignment of *At*TIG1 with *Cr*TIG1. For correction of the mature protein, 49 amino acids were removed from the N-terminal sequence with oligos 5′-GGAAGAGCTCATATGTCCCGTCTCTTCCAATCAGATAGTGT GCTACAGTTTGGTGGGAGGTTGAAGAAACCAATTAGCAGGCCTTTGGACATGTCTTGTGTCTCTAGAAAAATTGGATTTTTCGGA GATTTTATGTCACATGGTGGTAATTTTAGGCTATTC-3′ by site directed mutagenesis resulting in pFW136. The TF sequence from *E*. *coli* was amplified by PCR from DH5alpha genomic DNA with primers (5′-GGGGCATATGCAAGTTTCAGTTGAAACCAC-3′) and (5′-CCCCGGATCCTTACGCCTGCTGGTTCATC-3′) and cloned with NdeI and BamHI into pTyb21 (NEB) resulting pFW142. For heterologous protein production pFW115, pFW136 and pFW142 were synthesized in *E*. *coli* ER2566 and purified via chitin affinity resins according to the manufacturer’s instructions (NEB). All purified proteins were concentrated with Amicon Ultra-15 concentrator (Merck Millipore, Darmstadt) and purified over a Superdex 200 SEC column (GE healthcare) in 10 mM Tris pH 7.5 and 10 mM KCl buffer. For antisera production, rabbits were immunized with *Cr*TIG1 or *At*TIG1.

### Phylogenetic analyses and comparison of sequence similarity and identity

Sequences were derived from http://phytozome.jgi.doe.gov, NCBI and http://www.uniprot.org. Phylogenetic analyses were performed with sequences comprising the mature sequences lacking predicted transit peptides using the Phylogeny.fr pipeline^52^ implementing algorithms T-Coffee^53^, BioNJ^54^, and TreeDyn^55^ setting bootstraps values to 1000. Sequence similarity and identity was determined by http://imed.med.ucm.es/Tools/sias.html.

### Immunofluorescence microscopy

Cell fixation and staining was done as published before^56^. Primary antibodies were against *Cr*Tig1, RbcL, and PRPL1 in dilutions of 1:500, 1:2,000, and 1:1,000, respectively. The secondary fluorescein isothiocyanate-labeled antibody (Sigma-Aldrich) was applied in a 1:200 dilution. FISH probes were prepared according to^57^. After incubation with the secondary antibody, slides were washed in phosphate-buffered saline, and a drop of mounting solution containing DAPI (Vectashield; Vector Laboratories) was applied at the centre of each slide. Samples were analysed with a Leica TCS SP5 II confocal laser-scanning microscope (514-nm excitation and 500-, 592-, or 598–657 nm detection).

### Circular dichroism spectroscopy

CD spectra were recorded on a Chirascan-plus (Applied Photophysics) spectropolarimeter. All proteins were measured at a concentration of 0.1 mg/mL in 10 mM KCl, 10 mM Tris, pH 7.5 buffer. At fixed temperatures (20 °C), five scans between 190 nm and 280 nm were recorded with an optical pathlength of 0.1 cm, a step size of 1 nm, and a digital integration time of 1 s. All spectra were baseline-corrected. At the two minima at 222 nm and 208 nm, temperature scans were recorded from 20 °C to 94 °C at a heating rate of 1 °C/min and a digital integration time of 4 s.

### Thermal stability/melting point determination using Differential Scanning Fluorimetry

Protein thermal stability was measured at 5 mg/mL protein concentration in different buffer compositions using the Prometheus NT.48 (NanoTemper Technologies). Thermal unfolding was performed in nanoDSF grade high-sensitivity glass capillaries (NanoTemper Technologies) with a heating rate of 1 °C/min over a temperature range of 20–95 °C. Protein-unfolding midpoints (*T*_m_) were calculated from the first derivative of the ratio of intrinsic protein fluorescence emission intensities at 330 nm and 350 nm^58, 59^.

### Analysis of protein aggregates

*C*. *reinhardtii* cells were grown to logarithmic phase. For each sample, 3 × 10^8^ cells were harvested and resuspended in 50 mL fresh medium at the respective temperatures. After 45 min cells were harvested quickly and chilled on ice. Aggregate preparations were done as published previously^60^. In brief, cell pellets were resuspended in lysis buffer (20 mM NaP_i_ pH 6.8, 10 mM DTT, 1 mM EDTA, 0.25x proteinase inhibitor (Roche, cOmplete-EDTA-free)) and lysed by sonication (Bandelin Sonopuls UW 2200, Berlin). Cell debris was removed by centrifugation at 500 *g*, and supernatants were adjusted to same protein concentrations. As input control, “Total lysate” aliquots were removed and supplemented with 1 volume of sample buffer (125 mM Tris-HCl, pH 6.8, 20% glycerol, 4% SDS, 100 mM DTT, and 0.005% bromophenol blue). From remaining lysates, protein aggregates were isolated by repetitive centrifugation at 19,000 *g* and washes with 20 mM sodium phosphate pH 6.8 and 2% NP-40. The final pellet was dissolved in 6 M urea and supplemented with 1 volume of sample buffer before separation on SDS-PAGE, transfer to nitrocellulose membrane, and immunoblotting.

### RALS measurements

SEC coupled to RALS was performed on an OMNISEC system (Malvern Instruments, Worcestershire, UK) using a Superdex 200 Increase 10/300 SEC column (GE Healthcare). Prior to each run, the system was extensively equilibrated in running buffer (20 mM Tris, pH 7.5, 150 mM KCl). Protein concentrations were determined from online refractive index (RI) measurements assuming a d*n*/d*c* of 0.185 mL/mg. 50 µL of a calibration standard (BSA, Sigma, at 3 mg/mL) and samples (at 4 mg/mL) were injected twice. All experiments were conducted at 20 °C with a flow rate of 0.5 mL/min for 70 min. Molar masses were calculated as described by the manufacturer using OMNISEC v10 software.

### X-ray scattering experiments and data analysis

Small-angle X-ray scattering data were collected on the BM29 beamline^61^ at the European Synchrotron Radiation Facility (ESRF, Grenoble) with a Pilatus 1 M detector (16.9 cm × 17.9 cm) at a wavelength of 0.9919 Å (12.5 keV) and a sample-detector distance of 2.867 m corresponding to a *q*-range of 0.025–5 nm^−1^. For static measurements, five to eight different protein concentrations were measured with 1 s exposure times per frame and 10 frames per concentration (*n* = 3) at 20 °C. Scattering by proteins samples were measured in 20 mM Tris buffer, pH 7.5 supplemented with 150 mM KCl. Scattering by the corresponding buffers was measured before and after one run, averaged and subtracted from the protein scattering. BSA standards were used to calibrate the *I*(0) values and the scattering of pure water was used to calibrate the intensity to absolute units^62^. Prior to measurements, protein concentrations were determined from absorption at 280 nm (*A*_280_) using a NanoDrop One photometer (Thermo Scientific) and the corresponding molar coefficient calculated with ProtParam (www. expasy.ch).

For SEC high-performance liquid chromatography/small-angle X-ray scattering (HPLC-SAXS), an online HPLC system (Shimadzu, Japan) connected to the sample changer of the BM29 was used^63^. Per run, 400 µg protein (50 µL with 8 mg/mL protein concentration) was injected onto an ENrich SEC 650 10 mm × 300 mm column (BioRad) and separated at a flow rate of 0.75 mL/min at room temperature. 1500 frames with an exposure time of 1.5 s per frame were collected per run.

The data processing was done with the ATSAS 2.7.2 program package^64, 65^. The forward scattering *I*(0) and the radius of gyration *R*_g_ were evaluated with PRIMUS^66^ using the Guinier approximation assuming that for a spherical particles at very small angles (*s* < 1.3/*R*_g_) the intensity is represented by *I*(*s*) = *I*(0) exp[−(*sR*_g_)^2^/3]). The distance distribution function *p*(*r*) and the maximum particle dimension (*D*_max_) were obtained using *GNOM* ^67^.

Frames collected from static measurements were checked for radiation damage before averaging and buffer subtraction. Scattering curves from the concentration series were investigated individually for indications of aggregation and particle attraction or repulsion. Curves affected were excluded from further analysis. For the characterization of the oligomerization state, scattering curves (from *n* = 3) with same protein concentration were scaled and merged. For *ab initio* modelling, frames from different concentrations were merged. Frames of the HPLC-SAXS, which were automatically processed by EDNA, were checked and inspected with HDFview. Frames with a consistent *R*_g_ from the peak scattering intensity and good quality were scaled manually and averaged to yield a single frame. Buffer subtraction was applied using buffer frames before or after the protein peak avoiding over-subtraction with frames from the aggregate peak.

A first insight into the overall shape of the TIG1 proteins was given by the *AMBIMETER* tool. These models yielded a crude shape topology, which was further refined and supported by *ab initio* modelling. For the latter, 10–20 models were generated with *GASBOR* ^68^ or *DAMMIF* ^69^ and then averaged with *DAMAVER* ^70^. As a last refinement step, the *damstart* model generated with *DAMAVER* was refined with one cycle in *DAMMIN*. Protein structure modelling was performed with the SAXSTER server^71^ on the basis of all available full-length TF structures (i.e. from *E*. *coli*, *T*. *maritima* and *V*. *cholerae*) as templates or RaptorX^43^ algorithm based on the *Ec*TF structure. Superposition of *ab initio* models derived from SAXS data and 3D models were calculated with *SUPCOMB* ^72^ or *SUPALM* ^73^. Fits between the SAXS data and the structures were evaluated with *CRYSOL* ^74^ and *FoXS* ^75^ and superposed with the bead models using *SUPALM* ^73^. The models were improved using *SREFLEX* in rigid or flexible mode with domain partition^76^. To this end, sequences regions of the domains were determined from the RaptorX model in accordance with the domain annotation of the available TF structures (Supplementary Table S8). Additionally, theoretical scattering profiles from the models were generated and fitted against the experimental data with the FoXS Server^75^.

### Miscellaneous

Chloroplast and mitochondria were isolated from *C*. *reinhardtii* cells as described earlier^26^. SDS-PAGE was performed as published^77^. Antibodies described earlier were against HSP70B^48^, CGE1^78^, HSP90C^26^, mitochondrial carbonic anhydrase^79^, CytF^80^ and CF1β^81^. Antibody purification was described earlier^26^.

## Electronic supplementary material

Supplementary Information
