# Supplementary material for: Structural and molecular comparison of bacterial and eukaryotic trigger factors
Source: Sci Rep. 2017 Sep 6;7:10680. doi: 10.1038/s41598-017-10625-2 (PMC5587573; doi:10.1038/s41598-017-10625-2)

Supplementary Information

Structural and molecular comparison of bacterial and eukaryotic trigger factor

Fabian Ries[1§], Yvonne Carius[2§], Marina Rohr[1], Karin Gries[1], Sandro Keller[3], C. Roy D. Lancaster[2*], and Felix Willmund[1*]

[§] These authors contributed equally

Author's institution(s)/affiliation(s)

[1] Molecular Genetics of Eukaryotes, University of Kaiserslautern, Erwin-Schrödinger-Str. 70, 67663 Kaiserslautern, Germany

[2] Department of Structural Biology, Saarland University, Center of Human and Molecular Biology (ZHMB), Faculty of Medicine Building 60, 66421 Homburg, Germany

[3] Molecular Biophysics, University of Kaiserslautern, Erwin-Schrödinger-Str. 13, 67663 Kaiserslautern, Germany

* Corresponding authors: Felix Willmund (willmund@bio.uni-kl.de) and C. Roy D. Lancaster (Roy.Lancaster@structural-biology.eu)

Supplementary Tables

Table S1. Trigger factor sequences used for the phylogenetic tree in Figure 1d and Supplementary Figure S1.

Name[1]	Species	Gene Model	Accession Number (UniProt)[2]	Sequence Length (mature)[3]	Localisation[4]	Predicted Transit Peptide[4]
TF	Escherichia coli		P0A850	432	Cyt	-
TF	Vibrio cholerae		A0A0E4CAI9	433	Cyt	-
TF	Thermotoga maritima		Q9WZF8	425	Cyt	-
TF	Nostoc sp. strain PCC 7120		Q8YQX9	471	Cyt	-
TF	Synechocystis sp. strain PCC 6803		Q55511	471	Cyt	-
TF	Prochlorococcus marinus		A3PFE3	477	Cyt	-
TF	Synechococcus sp. strain WH8102	Gasu_56370	Q7UA35	472	Cyt	-
TIG1	Galdieria sulphuraria	1309	M2XTI8	468	CP	69
TIG1	Phaeodactylum tricornutum	4201	B7S404	579	CP	67
TIG1	Cyanidioschyzon merolae	Vocar.0013s0039 Cre01.g001750	M1UR28	464	CP	76
TIG1	Volvox carteri			486	CP	81
TIG1	Chlamydomonas reinhardtii	67252	A8JD56	481	CP	67**
TIG1	Coccomyxa subellipsoidea	61093	I0YR07	447	CP	57
TIG1	Micromonas sp. strain RCC299	35996	C1EBJ4	472	CP	39
TIG1	Ostreococcus lucimarinus	Pp3c11_10950V3	A4RSJ3	479	CP	54
TIG1a	Physcomitrella patens	Pp3c2_30270V3		532	CP	30
TIG1b	Physcomitrella patens	Pp3c14_20300V3	A9RQV7	524	CP	37
TIG2	Physcomitrella patens	GRMZM2G127393 AC203956.3_FGT004		182	CP	68
TIG1	Zea mays	GRMZM2G109526	K7TWH1	469	CP	59
TIG2a	Zea mays	GRMZM2G306172	K7VK28	412	CP	78
TIG2b	Zea mays	Seita.4G136300	B4FYP9	230	CP	19
TIG2c	Zea mays	Seita.2G272700		264	*	14
TIG1	*Setaria italica*	Seita.5G412100	K3XWC5	446	CP	65
TIG2a	*Setaria italica*	LOC_Os06g20320	K3ZWT0	186	CP	33
TIG2b	*Setaria italica*	LOC_Os09g34000	K3XLM0	164	*	65
TIG1	*Oryza sativa*	LOC_Os01g66980	A2YC62	469	CP	68
TIG2a	*Oryza sativa*	Bradi1g42820	A0A0P0XP17	122	CP	87
TIG2b	*Oryza sativa*		B8A7T9	147	*	82
TIG1	*Brachypodium distachyon*	Bradi4g35170	I1GYS7	461	CP	64
TIG2a	*Brachypodium distachyon Brachypodium*	Bradi2g57530	I1IRU4	157	*	41
TIG2b	*distachyon Brassica rapa*	Brara.B01334 Brara.D01836	A0A0Q3GIQ1	187	*	62
TIG1	*Brassica rapa*	AT5G55220		505	CP	42
TIG2	*Arabidopsis*		A0A078GVX1	145	CP	56
TIG1	*thaliana Arabidopsis*	AT2G30695	Q8S9L5	471	CP	76**
TIG2	*thaliana* Capsella	Cagra.2897s0023	Q945Q5	145	CP	54

TIG1	*grandiflora*	Cagra.6622s0002		492	CP	58
TIG2	Capsella grandiflora	Potri.015G065900		145	CP	54
TIG1	*Populus trichocarpa*	Potri.013G125600	B9IEG6	467	CP	61
TIG2a	*Populus trichocarpa*	Potri.013G125500	U5FY48	174	CP	74
TIG2b	*Populus trichocarpa*		U5FVR2	144	CP	66

[1] All protein sequences including an N-terminal ribosome-binding-domain, a peptidyl–prolyl isomerase domain and a C-terminal domain were termed TIG1, all protein sequences with only a ribosome-binding domain were termed TIG2.

[2] UniProt protein accession numbers are given only for sequences with a 100% match by BLAST search.

[3] Including N-terminal methionine for all sequences lacking a transit peptide.

[4] Localisation and transit-peptide length were predicted by TargetP and ChloroP[1,2].

* Localisation and transit-peptide length were predicted by TargetP and ChloroP but returned no clear result.

** Confirmed in this study.

Table S2: Molecular masses determined by multidetector SEC. Retention volume, molecular masses, and dispersity (i.e., mass-averaged molar/number-averaged molar mass) were calculated as described in Methods.

	Retention volume [mL]	Molar mass [kg/mol]	Polydispersity [M_w/M_n]
*Ec*TIG	13.55	79	1.01
*Cr*TIG1	12; 14.5	108; 55.6	1.001
*At*TIG1	14.3	63	1.002

Table S3: Structural parameters of *Ec*TF derived from different concentrations in static SAXS measurements (n = 3).

Conc. [mg/mL]	$I(0)$	R_g [Å]	Quality [%]	$I(0)$	D_{max} [Å]	R_g [Å]	Vp [Å^3]	MW [kDa] Vp	MW [kDa] $I(0)_{BSA}$	MW [kDa] $I(0)_{Water}$
	based on Guinier fit			calculated from *p(r)* function						
7.2	72.99 ± 0.02	34.7 ± 0.0	93	74.21	115	36.1	178680	105	72.77	70.12
3.8	73.06 ± 0.0	36.8 ± 0.0	74	73.40	112	37.3	186690	110	72.84	70.19
1.9	76.53 ± 0.04	37.5 ± 0.0	83	76.98	130	38.1	180570	106	76.30	73.53
1.0	74.24 ± 0.08	38.2 ± 0.0	82	74.37	133	38.5	159600	94	74.02	71.32
0.5	71.89 ± 0.11	37.6 ± 0.0	84	72.64	137	38.9	161740	95	71.67	69.07

Table S4: Structural parameters of *Cr*TIG1 derived from different concentrations in static SAXS measurements (*n* = 3).

Conc. [mg/mL]	*I*(0)	R_g [Å]	Quality [%]	*I*(0)	D_{max} [Å]	R_g [Å]	*Vp* [Å^3]	MW [kDa] *Vp*	MW [kDa] $I(0)_{BSA}$	MW [kDa] $I(0)_{Water}$
	based on Guinier fit			calculated from *p(r)* function						
8	62.02 0.03	47.1 ± 0.5	90	62.06	205	48.0	144200	85	61.83	59.58
4	56.36 ± 0.06	42.1 ± 0.4	84	56.34	160	42.7	129680	76	56.19	54.15
2	55.62 ± 0.04	41.1 ± 0.3	94	55.35	147	41.2	145050	85	55.45	53.43
1	52.10 0.08	38.6 ± 0.5	78	52.09	133	39.3	140230	82	51.94	50.05
0.5	50.98 ± 0.12	38.4 ± 0.7	79	50.96	127	39.0	118470	70	50.83	48.98

Table S5: Structural parameters of *At*TIG1 derived from different concentrations in static SAXS measurements (*n* = 3).

Conc. [mg/mL]	i(0)	R_g [Å]	Quality [%]	*I*(0)	D_{max} [Å]	R_g [Å]	Vp [Å^3]	MW [kDa] *V*p	MW [kDa] $I(0)_{BSA}$	MW [kDa] $I(0)_{Water}$
	based on Guinier fit			calculated from *p*(r) function						
8	58.33 ± 0.03	43.6 ± 0.2	91	58.58	198	44.8	154430	91	58.15	56.04
4	53.24 ± 0.05	39.5 ± 0.2	80	53.28	141	40.1	136910	81	53.08	51.15
2	51.92 ± 0.05	40.3 ± 0.4	94	51.28	131	39.0	143200	84	51.76	49.88
1	49.49 ± 0.07	36.8 ± 0.3	80	50.23	138	38.2	131700	77	49.34	47.45
0.5	48.73 ± 0.08	37.1 ± 0.3	85	48.93	135	37.9	110910	65	48.58	46.82

Table S6: Summary of SAXS data analysis and model refinement.

	Protein	Guinier fit		Final χ^2 (R-Factor)	Average NSD	χ^2 (*CRYSOL*)	χ^2 (*FoXS*)	NSD (SUPALM)
		Range	R^2	*DAMMIN* refined	10 *GASBOR* models	Final model (pre/post *SREFLEX*)		Final model against SAXS shape (pre/post *SREFLEX*)
Static SAXS	*Ec*TF	18-57	0.99973	n. d.	n. d.	n. d.	n. d.	n. d.
	*Cr*TIG1	43-63	0.99928	0.811 (0.0011)	1.747 ± 0.049	10.4/6.17	3.03/3.61	3.12/3.51
	*At*TIG1	18-63	0.99976	0.689 (0.0015)	1.729 ± 0.064	57.2/3.3	5.57/1.5	3.082.67
		Range	R^2	*DAMMIN* refined	20 *DAMMIF* models	Final model (pre/post *SREFLEX*)		Final model against SAXS shape (pre/post *SREFLEX*)
HPLC-SAXS	*Ec*TF	13-65	0.9995	n.d.	1.007 ± 0.07	n. d.	n. d.	n. d.
	*Cr*TIG1	30-76	0.9985	0.72 (0.0005)	0.731 ± 0.025	1.13	0.98/1.03	2.65/2.93
	*At*TIG1	19–73	0.9985	0.79 (0.0010)	1.05 ± 0.03	2.03	1.28/0.98	3.05/2.69

Table S7: Psi-Blast results of *Cr*TIG1 and *At*TIG1 amino acid sequences against Protein Data Base structures using BLASTP 2.2.26[3].

	*Cr*TIG1			*At*TIG1		
PDB (protein)	Score	Identity	Identities	Score	Identity	Identities
1T11 (*Vc*TF)	71.6	24 %	84/338	72.4	24%	82/338
2MLX (*Ec*TF)	70.5	21 %	102/467	77.0	21%	94/446
1W26 (*Ec*TF)	70.5	21 %	102/467	77.0	21 %	94/446
2VRH (*Ec*TF)	70.5	21 %	102/467	77.0	21 %	94/446
3GTY (*Tm*TF)	47.4	19 %	71/356	68.6	21 %	93/427

Table S8: Domain predictions of chloroplast trigger factor based on RaptorX and *I-TASSER* algorithm.

Protein	N-terminal domain	linker	PPIase domain	linker	C-terminal domain
*Cr*TIG1	Ala1-Asp137	Val138-Gly180	Phe181-Thr276	Leu277-Asn281	Asp282-Val481
*At*TIG1	Ala1-Asp137	Val138-Gly181	Ala182-Asp284	Leu285-Asp289	Asp290-Arg470

Supplementary Figure Legends

Figure S1: Phylogenetic analysis of full-length and truncated chloroplast trigger factors

Phylogram based on the amino acid sequences of full-length and truncated trigger factor versions (if present) from gram-negative bacteria (*Escherichia coli, Vibrio cholerae*), cyanobacteria (*Nostoc sp*. strain PCC 7120, *Synechocystis sp*. strain PCC 6803, *Prochlorococcus marinus, Synechococcus sp*. strain WH8102), diatom (*Phaeodactylum tricornutum*), the mature sequences of red algae (*Galdieria sulphuraria, Cyanidioschyzon merolae*), green algae (*Volvox carteri, Chlamydomonas reinhardtii, Coccomyxa subellipsoidea, Micromonas sp*. strain RCC299, *Ostreococcus lucimarinus*), moss (*Physcomitrella patens*), and land plants (*Zea mays, Setaria italica, Oryza sativa, Brachypodium distachyon, Populus trichocarpa, Brassica rapa, Arabidopsis thaliana, Capsella grandiflora*). Bootstrap values are given next to the nodes. For sequence information, see Supplementary Table S1. Bar indicates branch length.

Figure S2: Specificity of antibodies used for immunofluorescence

a. Specificity of antibodies used for immunofluorescence assays shown in Figure 2d. *Chlamydomonas* whole-cell lysate was separated by SDS-PAGE and immunoblotted with the indicated antibodies. **b.** Background control with secondary antibodies used for immunofluorescence (same intensity values as shown in Figure 2d).

Figure S3: Full elution profiles of SEC-RALS

Determination of conformational states of *Ec*TF, *Cr*TIG1, and *At*TIG1. 200 µg of protein was separated by size exclusion chromatography in a buffer containing 20 mM Tris, pH 7.5 and 150 mM KCl and measured at 90°. Here, the RALS signal was used to follow the elution. Average molar masses are indicated in blue for each graph (right ordinate).

Figure S4: Static SAXS measurements

Small angle X-ray scattering data were collected for various concentrations of trigger factor. **a.** Experimental SAXS profile from merged scattering curves of *Ec*TF are indicated by small circles. The red curve represents the fit by *GNOM*. Inset: Guinier plot of ln $I(s)$ *versus* s^2 obtained from *AUTORG.* Data points used by *AUTORG* are

labelled in blue. **b.** Kratky plot $s^2I(s)$ *versus* s^{-1}. **c.** Corresponding *p(r)* function as calculated from the experimental scattering curves using the *GNOM*. a.u. is arbitrary units. **d.** and **e.** Experimental SAXS profile of *Cr*TIG1 and *At*TIG1 (in grey) and the best *FoXS* fit (in green).

Figure S5: HPLC-SAXS measurements

Small-angle X-ray scattering data were collected by size-exclusion high-performance liquid chromatography using a ENrich SEC 650 column online with small-angle X-ray scattering (HPLC-SAXS) with 400 µg of the respective protein. **a.** Elution profiles of SEC-SAXS runs, represented by $I(0)$ and R_g for the frame for which these could be determined by *AUTORG*. Highlighted in grey are the frames used for averaging. **b.** Experimental SAXS profile of *Ec*TF is indicated by small circles. The red curve represents the fit by *GNOM*. Inset: Guinier plot of ln $I(s)$ *versus* s^2 obtained from *AUTORG.* Data points used by *AUTORG* are labelled in blue. **c.** Corresponding *p(r)* function as calculated from the experimental scattering curves using the *GNOM*. a.u. is arbitrary units. **d.** and **e.** Experimental SAXS profile of *Cr*TIG1 and *At*TIG1 (in grey) and the best *FoXS* fit (in green).

Figure S6: Global shape of chloroplast trigger factor

Ab initio modeling from SAXS data. Shape topology and low-resolution bead models of *Cr*TIG1 and *At*TIG1 from the static SAXS and HPLC-SAXS experiments were generated using *DAMMIN* on the basis of 10 averaged *GASBOR* models for static or 20 *DAMMIF* models for HPLC-SAXS experiments, respectively*.* The bead models were superimposed by *SUPALM* on the RaptorX (for *Cr*TIG1) high-resolution model or the improved models by *SREFLEX* (for *At*TIG1). Secondary structures were colored in yellow (β-sheets), red (α-helix), and green (random coil). Models are shown in four different angles.

References

1 Emanuelsson, O., Nielsen, H., Brunak, S. & von Heijne, G. Predicting subcellular localization of proteins based on their N-terminal amino acid sequence. *J Mol Biol* **300**, 1005-1016, doi:10.1006/jmbi.2000.3903 (2000).

2 Emanuelsson, O., Nielsen, H. & von Heijne, G. ChloroP, a neural network-based method for predicting chloroplast transit peptides and their cleavage sites. *Protein Sci* **8**, 978-984, doi:10.1110/ps.8.5.978 (1999).

3 Altschul, S. F. *et al.* Gapped BLAST and PSI-BLAST: a new generation of protein database search programs. *Nucleic Acids Res* **25**, 3389-3402 (1997).

Supplementary Figure S1

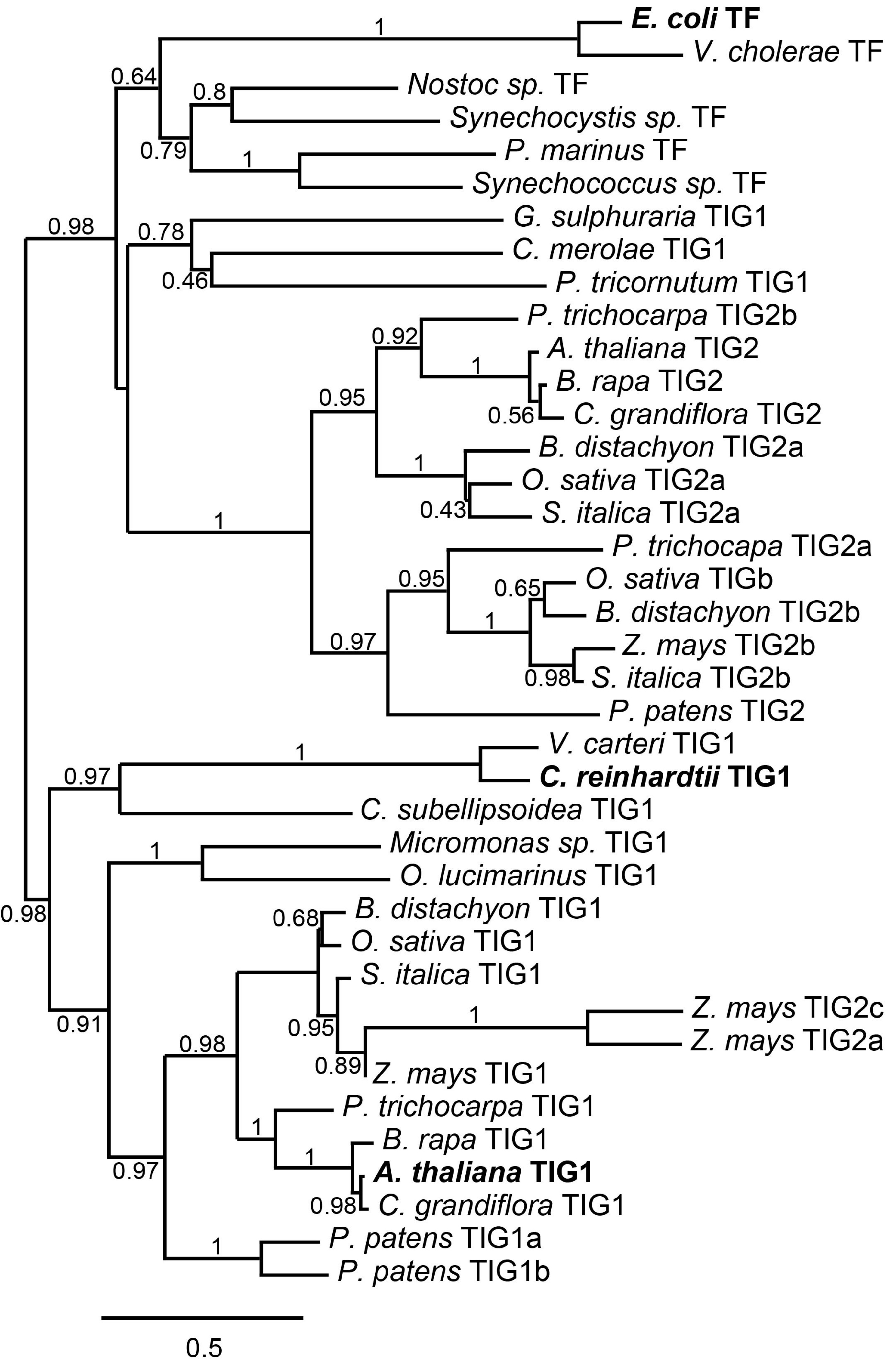

Supplementary Figure S2

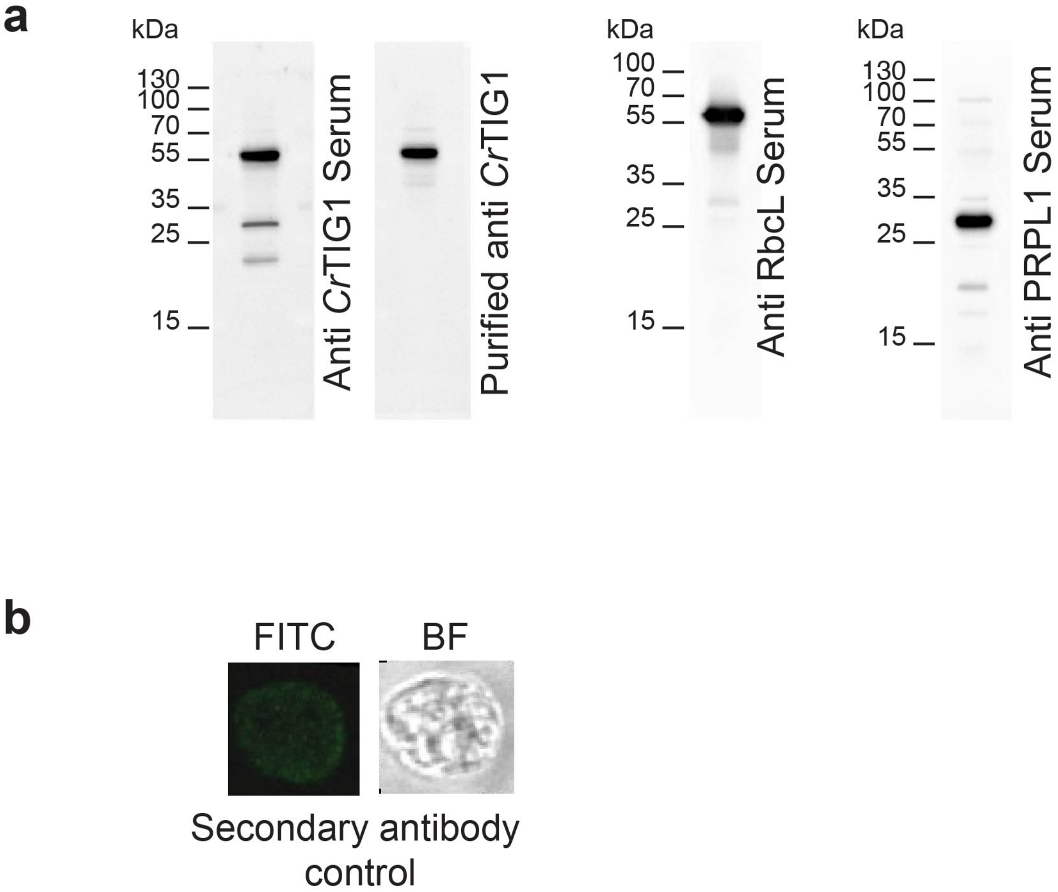

Supplementary Figure S3

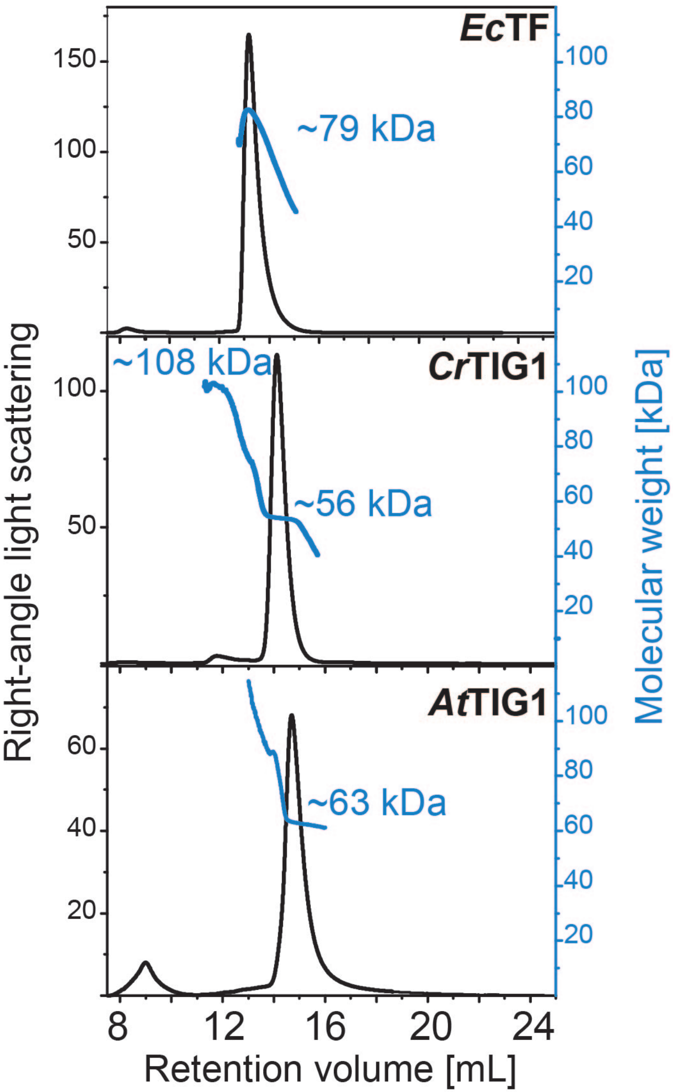

Supplementary Figure S4

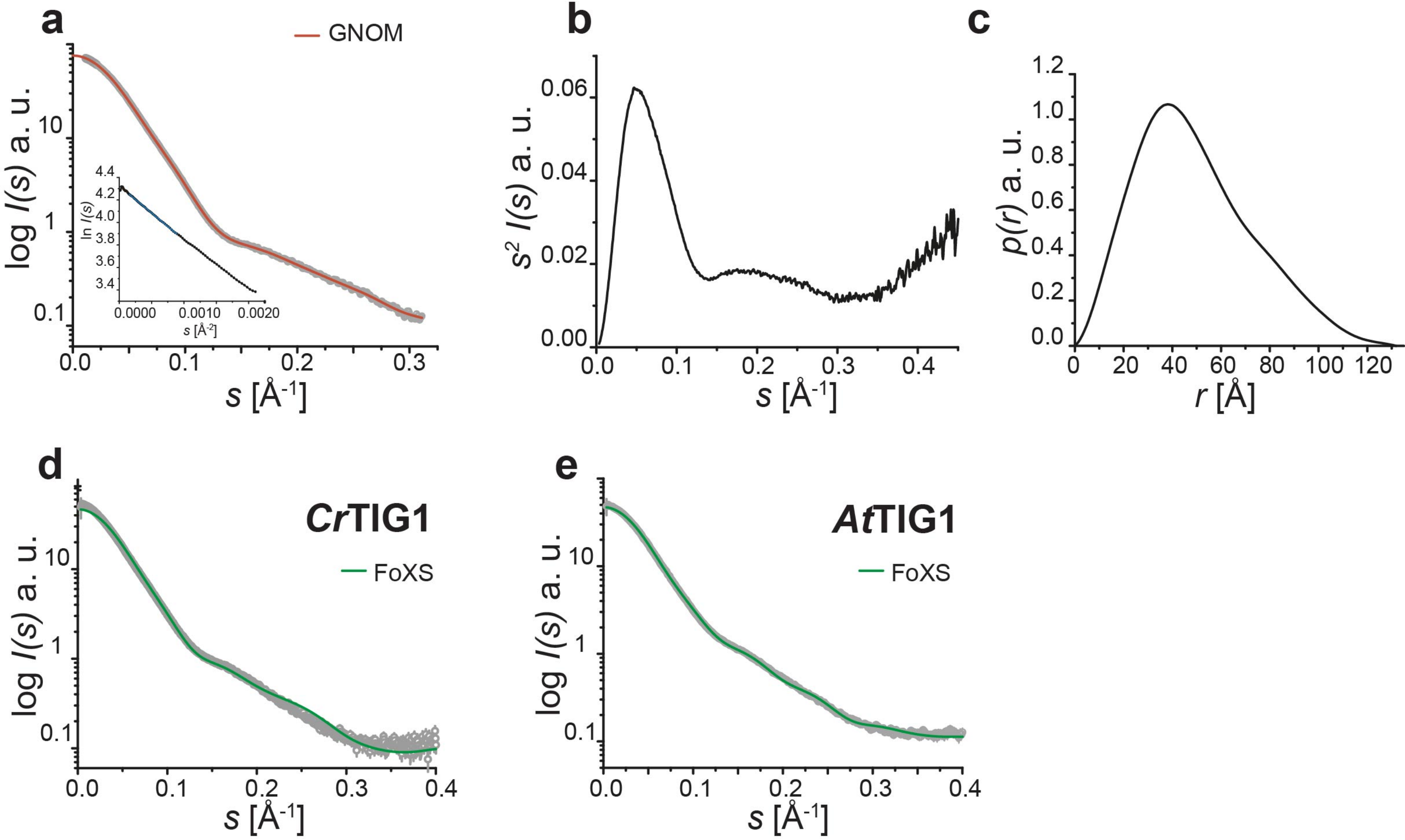

Supplementary Figure S5

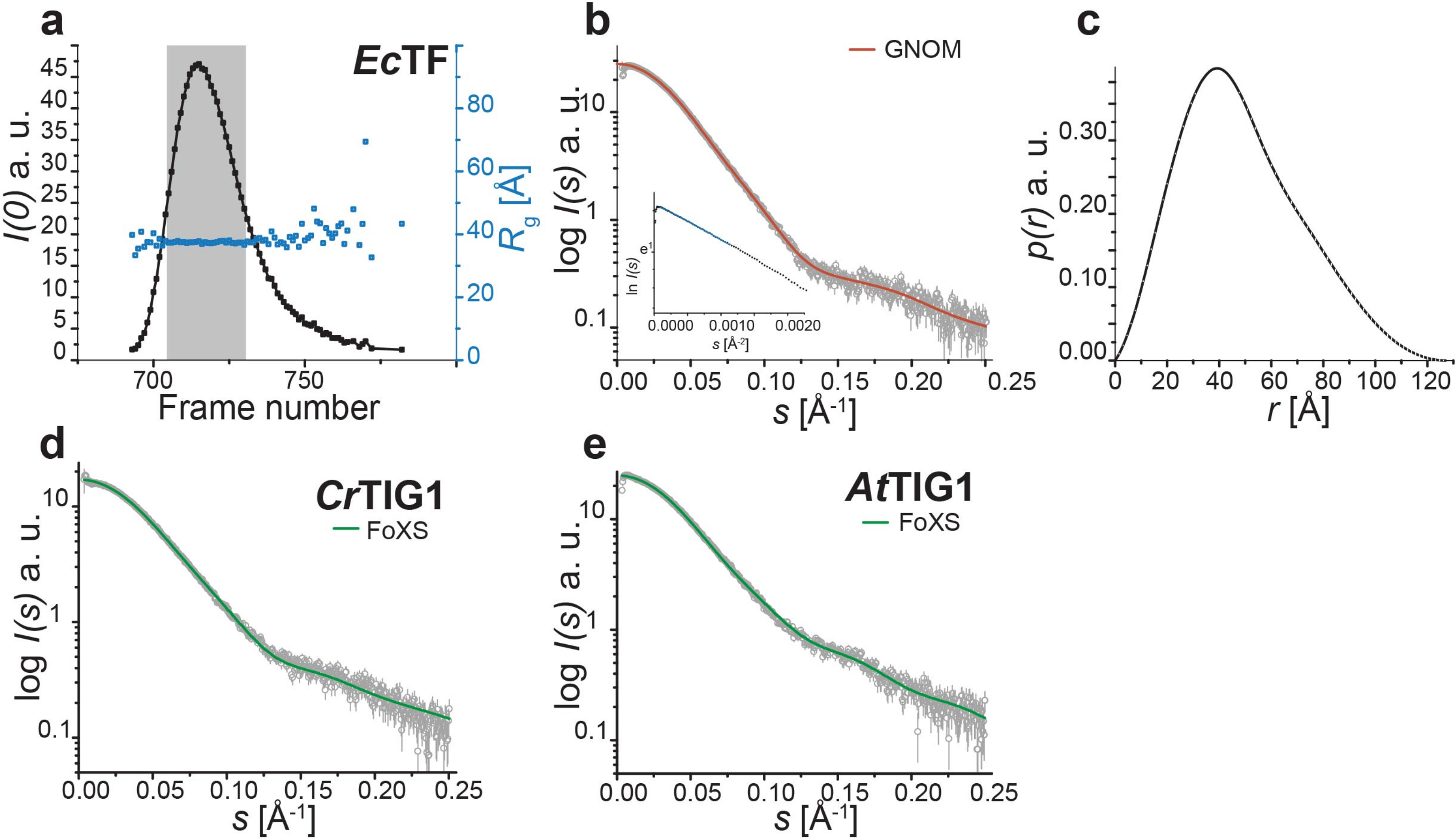

Supplementary Figure S6

*Cr*TIG1 HPLC *DAMMIN* vs. RaptorX

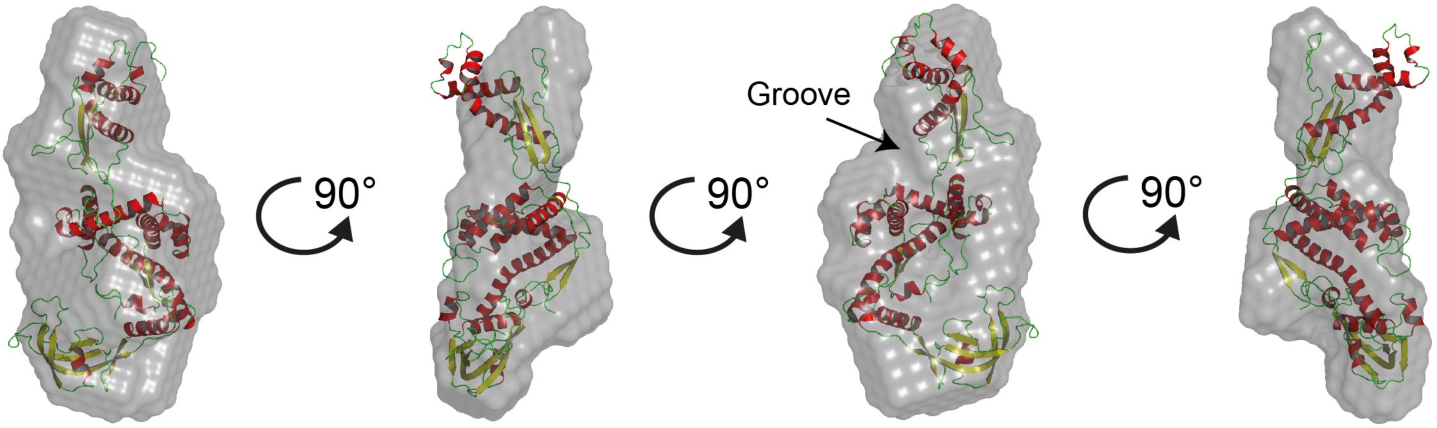

*Cr*TIG1 static (*GASBOR*) *DAMMIN* vs. RaptorX

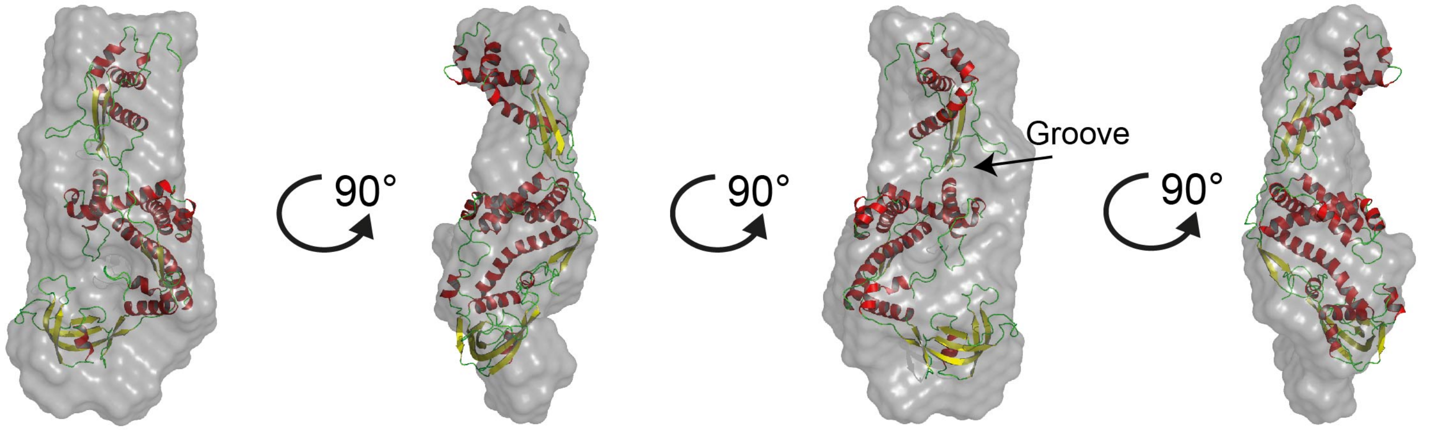

*At*TIG1 HPLC *DAMMIN* vs. *SREFLEX*

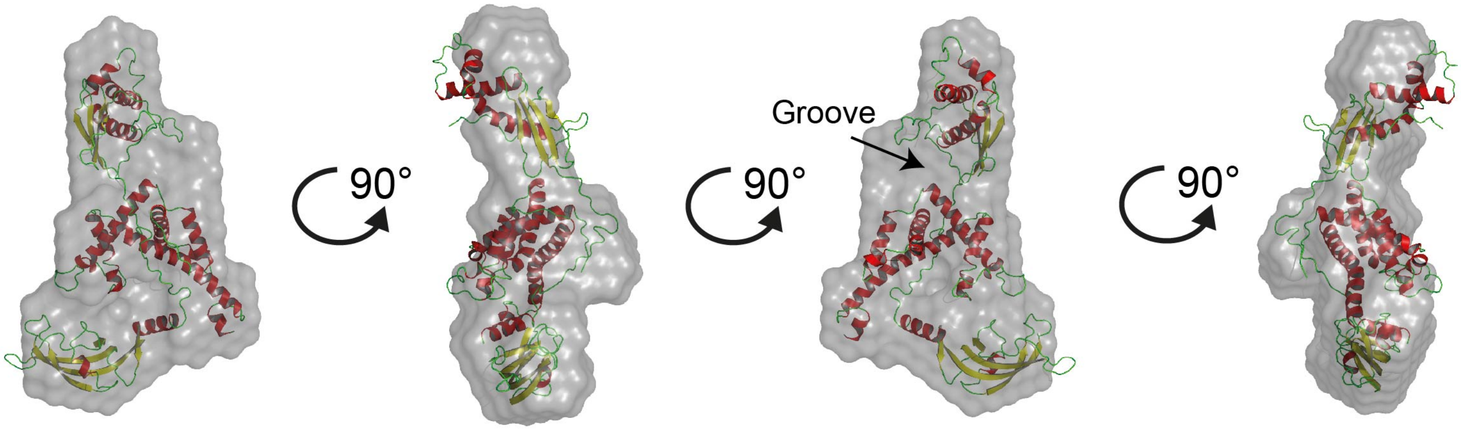

*At*TIG1 static (*GASBOR*) *DAMMIN* vs. *SREFLEX*

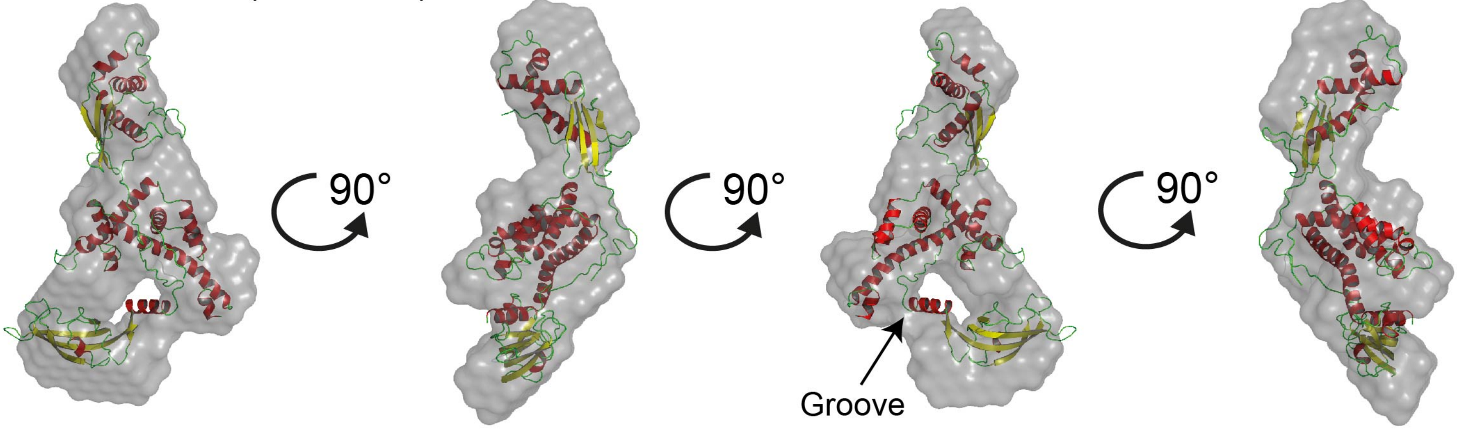

Supplement: Supplementary file 1 — Supplementary Information [file 41598_2017_10625_MOESM1_ESM.pdf]
